# Developing a toolkit for engagement practice: sharing power with communities in priority-setting for global health research projects

**DOI:** 10.1186/s12910-020-0462-y

**Published:** 2020-03-14

**Authors:** Bridget Pratt

**Affiliations:** Centre for Health Equity, School of Population and Global Health, University of Melbourne, 207 Bouverie St Street, Carlton, VIC 3053 Australia

**Keywords:** Ethics, Power, Global health, Engagement, Participation, Justice, Priority-setting, Research

## Abstract

**Background:**

Communities’ engagement in *priority-setting* is a key means for setting research topics and questions of relevance and benefit to them. However, without attention to dynamics of power and diversity, their engagement can be tokenistic. So far, there remains limited ethical guidance on how to share power with communities, particularly those considered disadvantaged and marginalised, in global health research priority-setting. This paper generates a comprehensive, empirically-based “ethical toolkit” to provide such guidance, further strengthening a previously proposed checklist version of the toolkit. The toolkit places community engagement and power-sharing at the heart of priority-setting for global health research projects.

**Methods:**

A two part method was used to generate a revised toolkit. Part one was conceptual, consisting of novel analysis of empirical data (previously collected as part of the same overall project) to identify additional concepts relevant to power-sharing between researchers and communities in global health research priority-setting. Part two was empirical, seeking feedback on the initial checklist version of the toolkit in interviews with researchers, ethicists, community engagement practitioners, and community organisation staff.

**Results:**

The conceptual process identified two additional components of engagement and six additional features that affect who defines, who participates, and who is heard in research priority-setting. New ethical considerations related to sharing power in global health research priority-setting are articulated in relation to those components and features. Interviewees provided suggestions for revising the toolkit’s content and language. The implications of these suggestions and the analytic process for the toolkit are described.

**Conclusions:**

The resultant toolkit is a *reflective* project planning aid for researchers and their *community* partners to employ before priority-setting is undertaken for global health research projects. It consists of three worksheets (to be completed collectively) and a companion document detailing how to use them. It is more comprehensive than the initial toolkit, as worksheet questions for discussion cover all phases of priority-setting.

## Background

Global health research priority-setting is dominated by funders and researchers, often from high-income countries. Communities, particularly those considered disadvantaged and marginalised, rarely have a say in setting the research topics and questions of global health research projects. Here, global health research is defined as research addressing health problems worldwide, especially those of the most marginalised, who live primarily (but not exclusively) in low and middle-income countries.

As a matter of health and social justice, bioethicists have argued that global health research should improve the health and well-being of those considered disadvantaged and marginalised and foster their participation in decision-making about its conduct [4, 17, 26]. This entails engaging such communities throughout research projects, including when setting research topics and formulating research questions [21, 26]. Their engagement in priority-setting is a key means for setting global health research priorities that matter to them. It can draw out their ways of knowing, thereby promoting cognitive and epistemic justice and maximising the social knowledge used to set research agendas [13, 21]. In this paper, the community is defined broadly to encompass policymakers, local leaders, community organisations, health providers and managers, patients, their families, and other community members [22].

However, without attention to dynamics of power and diversity, communities’ engagement can be tokenistic. Voices are excluded from priority-setting, especially those already marginalised by their societies’ institutions and norms. Existing evidence confirms that, for example, being *female*, being *poor*, having *little formal education*, living with a *disability*, and/or belonging to minority *ethnic groups* means certain individuals are listened to less or not at all in health priority-setting [27]. Where voices from marginalised communities aren’t heard, global health research projects won’t prioritise the key problems they face in accessing and affording health care and services. Projects are then much less likely to generate evidence that will improve health care and systems for them.

What is needed to ensure marginalised communities’ voices are raised and heard in agenda-setting for global health research projects? So far, there remains limited ethical guidance on this matter. It is unclear how priority-setting should be designed so that that their knowledge and voices are visible in its outputs. A significant amount of existing literature explores the concept of participation in decision-making in contexts of power disparities, spanning disciplines like political philosophy, ethics, development studies, health policy, and community-based participatory research (see [1, 2, 6–8, 11, 12, 16, 18–20, 28–30]). That body of work largely does not consider the participation of marginalised communities in the context of *health research priority-setting*. Existing ethical guidance on community engagement in global health research (e.g. CIOMS, UNAID, and NIAID guidelines) generally does not discuss how power imbalances should be dealt with, including those existing within communities [26]. Unsurprisingly then, tools and resources have not been created to help researchers and their partners share power with communities in their priority-setting practice.

### The research program

To address this gap in guidance and resources, a program of empirical ethics research is being undertaken. It aims to characterise the ethical considerations related to sharing power that should be taken into account when engaging communities in priority-setting for global health research projects and to develop guidance on how to address them. It further aims to create a resource—an “ethical toolkit”—for researchers and their partners to use in their practice that incorporates the findings of that research. The toolkit helps them design priority-setting processes that meaningfully include the communities impacted by their projects. It places community engagement and power-sharing at the heart of global health research priority-setting in order to make the health needs and knowledge of communities, particularly those considered disadvantaged and marginalised, more visible in research projects’ topics and questions.

The research program has employed an empirical reflective equilibrium approach [9, 15] to develop the ethical toolkit. These methods were selected because they comprise a robust methodology for developing ethical guidance that is informed by both theory and practice [10]. Traditionally, reflective equilibrium entails working back and forth between existing theoretical considerations (intuitions, moral principles, theories) and new ones, revising and refining until coherence is achieved [3]. In empirical ethics, it involves testing theoretical considerations against information from practice—namely, the considered judgements of people who perform or have significant experience with the studied practice [10].^1^

To identify theoretical considerations, publications on participation in contexts of power disparities were analysed to deconstruct the concept of engagement into its components and their associated sites of power [23]. The publications spanned six bodies of literature: development studies, political philosophy, health priority-setting, public deliberation, community-based participatory research (CBPR), and patient/consumer/user/community engagement/involvement in research. Their thematic analysis identified four *components of engagement*—who initiates, for what purpose, who participates, and how they participate—and *sites of power* (features of decision-making processes that affect who sets up the processes, who participates in them, and whose voices are reflected in their outputs) related to each of them [23]. Ethical considerations related to sharing power at each site were characterised. That conceptual work is comprehensively described in Pratt [23] and an initial version of the toolkit was developed based on it.

The initial toolkit comprised a checklist with items corresponding to the identified ethical considerations. That version indicated whether affirmative answers to each item were ethically ideal, preferable, minimally acceptable, or problematic. After answering all the items in the checklist, it was recommended that researchers and their partners modify their priority-setting processes’ design in order to achieve as many ethically ideal or ethically preferable answers as possible and as few (if any) ethically problematic answers [23]. The initial version of the toolkit is provided in Pratt [23].

To collect information from practice, the next step was to gather data on the following types of key informants’ experiences and perspectives:

- health researchers who engage communities, especially those considered disadvantaged and marginalised, in their studies,
- health researchers with experience in health research priority-setting at national level,
- ethicists with expertise in community engagement and/or research priority-setting,
- community engagement practitioners, and
- staff of community organisations (COs) who work with marginalised communities and have partnered with researchers to conduct health research.

This empirical work had two aims. First, to explore key informants’ perspectives on what is necessary to share power with communities, especially those considered disadvantaged and marginalised, in research priority-setting. Second, to obtain their suggestions for improving the preliminary toolkit. The findings related to the former aim are reported in Pratt [24, 25]. Briefly, thematic analysis identified categories corresponding to components of engagement and sub-categories corresponding to different sites of power. Interviewees described ways of sharing power at those sites. The findings related to the 2^nd^ aim are reported in this paper.

### The contribution of this paper

The purpose of this paper is to generate a more comprehensive and robust version of the ethical toolkit. Two key activities were undertaken to produce a revised toolkit—one conceptual and one empirical. The conceptual component had four main tasks:

1. To identify concepts from already published empirical work [24, 25] that aren’t in the already published conceptual work [23].
2. To use these concepts to characterise new ethical considerations related to power-sharing in global health research priority-setting.
3. To identify concepts from already published empirical work [24, 25] that are also found in the already published conceptual work [23].
4. To use data from the empirical work to refine ethical considerations articulated in the initial ethical toolkit that are related to these shared concepts.

The empirical component had one main task:

1. To report previously unpublished data on how to improve the toolkit gathered during interviews with researchers, ethicists, community engagement practitioners, and community organisation staff.

The paper reports the outputs of the conceptual and empirical components and discusses their implications for the ethical toolkit. The resultant version of the toolkit is described and then provided in Additional Files 1, 2, 3, 4, 5 and 6. It comprises a *reflective* project planning aid for use by researchers and their community partners (e.g. health care providers, policymakers, community organisations) before priority-setting is undertaken for a global health research project. It consists of three worksheets with open-ended questions for discussion (to be completed collectively), rather than a checklist, and a companion document detailing how to use the worksheets.^2^

## Methods

A two-part methodology was employed to generate a more robust version of the ethical toolkit. Part one comprised an analytic process using previously published data from the overall research program. First, components of engagement and sites of power from already published conceptual work [23] were compared to those from already published empirical work [24, 25]. Where additional components of engagement and sites of power were identified, conceptual work, drawing on power-sharing strategies discussed by interviewees, was performed to derive ethical considerations relevant to them. Where previously identified components of engagement and sites of power were identified, ethical considerations from the initial toolkit were examined in light of interviewees comments and refined.

Part two entailed seeking feedback on the initial version of the toolkit in interviews with key informants (researchers, ethicists, community engagement practitioners, CO staff) and one focus group with nine ethicists from the UK.^3^ In total, ten men and nineteen women were interviewed. Twelve interviewees live in Africa, nine in Australia, five in the UK or Europe, one in the USA, one in Latin America, and one in Asia. Nineteen were employed by universities or research institutions, four by non-governmental organisations, five by COs or civil society organisations, and one by a health organisation focused on the African region. There was overlap between types of key informants, with many interviewees meeting criteria of two or occasionally three of the categories. Interviewees in the researcher category had expertise conducting clinical, health systems, public health, development, and biomedical research. Written informed consent was obtained from all participants.

Interviewees and focus group members were asked to describe their main suggestions for improving the checklist version of the toolkit, which they had been provided with at least 1 week prior to interview. Interviews and the focus group discussion were approximately 30–75 min’ duration. They were transcribed verbatim and thematic analysis was undertaken by two coders in the following five phases: initial coding framework creation, coding, inter-coder reliability and agreement assessment, coding framework modification, and final coding of entire dataset [5, 14]. Once the coding framework was finalised, all 30 transcripts were re-coded using NVivo Version 11. Thematic analysis identified two main themes: suggestions for improving the toolkit’s content and suggestions for improving its language.

## Conceptual component results

### Comparing concepts identified by prior conceptual and empirical work

**Fig. 1:**
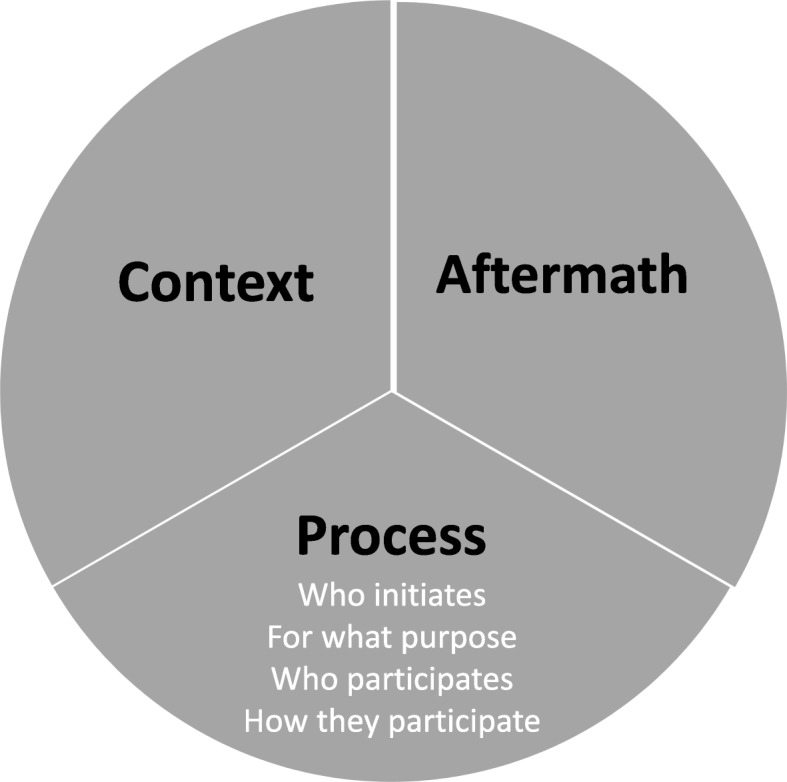
Components of engagement

Of the three components of engagement discussed by interviewees, two—context and aftermath—were not identified by the published conceptual work. Components of engagement previously identified by that conceptual analysis—who initiates, for what purpose, who participates, how they participate—were encompassed by the third component discussed by interviewees: process (Fig. 1). Context encompasses features of the research setting, team, and community of focus that can either facilitate or obstruct power-sharing. They include researchers and community members’ capacities, researchers’ degree of embeddedness in the community, their relationships, and existing norms and institutions within the community or country [24]. The temporal themes of process and aftermath encompass the sites of power that exist during and after global health research priority-setting [24].

Interviewees also described six sites of power—ground rules, transparency, respect, space, community assets, and accountability—not captured in the published conceptual work (Table 1). (This, however, does not mean these sites of power are not identified in any existing literature. Only that they were not found in the literature reviewed during the conceptual work.) Interviewees’ further identified seven sites of power that were also captured by the published conceptual analysis: leadership, framing, level of participation, stage of participation, representation, having voice, and mass (Table 1).

**Table 1:** Comparison of sites of power described by interviewees relative to those described in the literature

Component of engagement	Sites of power-sharing identified by **interviewees**	Sites of power-sharing identified by **both**	Sites of power-sharing identified in **the literature**
**PROCESS-***During* global health research priority-setting	**Ground rules** The rules under which health research priority-setting is undertaken. They specify who can and cannot be present, who can speak and when, how different individuals’ views are used, and how a decision or closure is reached.	**Leadership** Who takes the lead on key aspects of research priority-setting: planning, implementing, and ensuring outputs are fed back and used.	**Goal of engagement** The reason(s) for engaging community members in global health research priority-setting. Can be instrumental (i.e. as a means to another goal) and/or transformative (i.e. to generate empowerment).
	**Transparency** Whether there is openness and honesty about any constraints surrounding the priority-setting process, the ground rules for priority-setting, and what happens after priority-setting.	**Framing** What issues can be brought into the priority-setting space and what issues are not allowed; What information is presented or shared with participants at the start of the priority-setting process.	**Range** Whether community participants span a wide spectrum of relevant roles and demographics. Relevant roles could be: patients, families and carers, providers, purchasers, payers, policymakers, and product makers. Ensuring the presence of marginalised groups is also key to achieving range.
	**Space** The physical setting in which health research priority-setting is undertaken.	**Stage of participation** When community members are allowed to participate in the health research priority-setting process.	
	**Respect** Whether interpersonal and cultural respect are shown for community participants.	**Level of participation** The mode(s) of participation assumed by community participants during health research priority-setting.	
	**Community assets** Features of a community or a community organisation partner that can be drawn upon to support the representation and voices of its members, esp. those considered marginalised, during health research priority-setting.	**Representation** Whether community members are represented during health research priority-setting. Encompasses considerations related to the channel of representation and the genuineness of representation.	
		**Having voice** Whether community participants are able to speak and be heard during health research priority-setting. Encompasses considerations related to facilitation, documentation, and synthesis of global health research priorities.	
		**Mass** The numbers of participants representing powerful versus less powerful community members.	
**AFTERMATH-***After* global health research priority-setting	**Accountability** Responsibilities of researchers, research institutions, and community members after priority-setting.		

### Characterising additional ethical considerations^4^

#### Context considerations

In relation to context, interviewees highlighted that having COs as research partners can facilitate power-sharing with marginalised and disadvantaged communities, particularly where COs have certain values, capacities, and social standing in their community. It is important to select CO partners with some or all of the following features: social justice and equity as guiding values, local staff, peer-led community groups, strong networks within their community, experience doing grassroots work and outreach in vulnerable communities, and non-elite status [24]. A key ethical consideration before commencing community engagement in global health research priority-setting is then: *Does your research team include a research partner(s) and a community partner(s) who can access a community that is considered disadvantaged or marginalised?*

Interviewees also distinguished between long-term and oasis engagement. Oasis engagement was defined as researchers (from within the host country or from other countries) initiating a brand new set of relationships with community members as part of a single project. Long-term engagement meant researchers had worked and spent time in a research setting for many years, building relationships and conducting studies where community members’ ideas and views were sought [24]. Where such embedded work occurred over an extended period of time, the health research priorities of the community would have likely emerged. It is, therefore, essential to ask: *Have the community’s health research priorities, including those of the disadvantaged or marginalised, already been voiced?* If they have, it may not be necessary to undertake priority-setting exercises for new projects. Doing so, in fact, might comprise a waste of resources that could alternatively be spent on other research activities or engagement in other phases of the research process. The costs and benefits of undertaking engagement in research priority-setting in contexts of long-term engagement should be assessed. In cases where the costs outweigh the benefits and/or the benefits are small, engaging community members in priority-setting may not be the ethical course of action.

Where community health research priorities have not been voiced, it is important to ask: *Do the foundations for sharing power in priority-setting exist with this community?* Interviewees identified several contextual features that can facilitate or obstruct meaningful engagement in global health research priority-setting. To briefly summarise, facilitating features (or foundations) for power-sharing between researchers and communities included: community members’ understanding of research, health systems, and priority-setting; community members’ reflective knowledge; researchers’ personal and professional qualities; researchers’ knowledge of the community; and trusting relationships. Obstructive features included: funding constraints, unequal power dynamics, and the given community having been previously exploited by researchers [24].

The foundations for power-sharing are much more likely to be absent or inadequate in contexts of oasis engagement or where the research team has not worked with the given community for very long. To determine if they exist in a particular setting, the first step is to assess which facilitating and obstructive features are present and which are not. The next step is to consider what missing facilitating features can feasibly be built and what obstructive features can feasibly be mitigated. Where it is not possible to build or mitigate many of the features, proceeding with engagement in priority-setting may not be the ethical course of action in the short term.

#### Process and aftermath considerations

In relation to process and aftermath, six additional sites of power in global health research priority-setting were identified: ground rules, transparency, respect, space, community assets, and accountability. For each, ethical considerations were derived in light of the strategies interviewees described as being essential for sharing power with communities, especially those considered disadvantaged and marginalised, at the site. What *ground rules* are set for a priority-setting process can have a significant impact on who is present and whether their voices can be raised and heard. Ground rules specify who can and can’t be participants, who speaks, how different individuals’ views are used, and how a decision or closure is reached. Sharing power means that the terms under which global health research priority-setting is undertaken are not set by researchers alone. Ideally, CO staff and/or community members will be part of drafting ground rules as opposed to solely commenting on rules drafted by others [24]. A key ethical consideration is then: *Will you involve community members in developing and approving the ground rules for priority-setting?* Sharing power with those considered disadvantaged and marginalised, in particular, further calls for adopting specific ground rules designed to ensure their presence and voice. Examples include rules that all participants have an equal right to speak and that all ways of speaking (e.g. storytelling, narrative) are equally valuable and valid [24]. This means another key ethical consideration is: *What ground rules will you include to ensure those considered marginalised aren’t silenced during priority-setting?*

*Transparency* is important for building community members’ trust in the research team and the legitimacy of the global health research priority-setting process. Ensuring the ground rules of the priority-setting process are disclosed and explained to community members can promote their understanding of how the process will work and thus their capacity to raise their voices. Transparency can also help prevent community members from developing unrealistic expectations about what the priority-setting process can deliver. Where there is not an open scope to set research topics and questions with community members, it must be made very clear to participants that not all health research topics and questions can be raised [24]. Doing so means clarifying what topics are off the table and explaining why that is *before* starting priority-setting. Key ethical considerations for global health research priority-setting are then: *How will ground rules be clearly communicated to participants in priority-setting? Will it be made clear to participants that not all research topics can be raised during the priority-setting process and why that is?*

Demonstrating *respect* is a key way to promote the presence and voices of community members being raised, including those considered disadvantaged and marginalised, in global health research priority-setting. Two forms of respect were described as essential to sharing power: interpersonal and cultural [24]. An important ethical consideration for global health research priority-setting is: *How will you demonstrate respect to community members and their culture during priority-setting?* Demonstrating cultural respect (in part) means recognising and showing consideration for local power dynamics as much as possible [24]. Thus, another key ethical consideration is: *How will you respect local power dynamics while ensuring those considered marginalised within communities can raise their voice and be heard?* Achieving the latter may mean disrupting local power dynamics.

The physical *spaces* selected for global health research priority-setting can promote or obstruct the presence and voices of communities. Where spaces are physically difficult for community members to access, it can result in their not being present for priority-setting. Where spaces are imbued with certain norms, behaviours, and languages, people favoured by those norms or more practiced in those behaviours and languages will dominate priority-setting [24]. As such, community members will be more likely to raise their voices during priority-setting processes held in spaces within the community, as opposed to at research institutions or at the Ministry of Health. Yet even local spaces can be characterised by exclusionary norms, for example, where minorities are simply informed and generally do not have a say. Priority-setting using such spaces will then be characterised by minority groups being present but rarely speaking. An important ethical consideration when selecting spaces to hold global health research priority-setting is then: *Is that space physically accessible, safe and not imbued with norms that silence those considered marginalised?*

Communities have *assets* that can facilitate power-sharing with those considered disadvantaged and marginalised in health research priority-setting. These assets encompass physical and human resources, information and skills, networks, and participatory structures. Community champions, for example, can facilitate the recruitment of the marginalised and disadvantaged to priority-setting because they are knowledgeable about their community and the diversity within it. Such champions are trusted by community members and their endorsement of researchers can promote recruitment of those who would otherwise not be engaged [24]. A key ethical consideration for global health research priority-setting is then: *What assets within the community can be used to help recruit its members, especially those considered disadvantaged or marginalised, and to bring out their voices during priority-setting?*

Finally, accountability is important for ensuring community members’ voices are heard and building the legitimacy of the priority-setting process. It means that participants and the wider community hold the research team to a set of standards, judge whether the research team has fulfilled its responsibilities, and take measures if they determine that these responsibilities have not been met. Three responsibilities of the research team were identified as: 1) feeding back, 2) acting on research priorities, and 3) evaluation. Feeding back means sharing resultant research priorities with participants in the process and giving them an opportunity to comment on the priorities [24]. An ethical consideration for global health research priority-setting is then: *How will you feed back the final research topic and questions to community members, including those considered to be marginalised, after priority-setting?* Achieving action on the research priorities ensures that community members’ voices are heard. It means that research projects focusing on their priorities are funded and performed [24]. Another ethical consideration related to accountability is: *How will you act upon the final research topic and questions?* Finally, accountability as evaluation means developing benchmarks for power-sharing in priority-setting and assessing whether or not they have been met. Benchmarks could include achieving a diversity of participants and getting the voices of those considered disadvantaged heard [24]. This demands considering: *How are you and community members going to evaluate their engagement in the priority-setting process?* Where evaluations demonstrate that engagement falls short in terms of power-sharing, it then raises questions about whether it is ethical to take resultant research priorities forward.

### Refining ethical considerations

For each site of power described in both the previously published conceptual and empirical work, the ethical considerations in the initial toolkit [23] were taken as the starting point and examined in light of interviewees’ descriptions of what is needed to share power with marginalised communities at them. This analysis led to three types of revisions being made. First, where interviewees described key aspects of research practice at a site of power that weren’t captured in the prior conceptual analysis, ethical considerations’ content was revised to reflect their insights. In relation to having voice, interviewees identified facilitation as being where power dynamics are managed when engaging communities in research decision-making; they noted that having a local person act as facilitator was important to sharing power with community members [25]. Thus, ethical considerations related to power dynamics in deliberative spaces in the initial toolkit were replaced with ethical considerations related to facilitation (Table 2).

**Table 2:** Generating ethical considerations for the revised toolkit in relation to sites of power identified by both conceptual and empirical analysis

Site of power	Initial toolkit version of ethical consideration	Revised toolkit version of ethical consideration
Representation	1. Particular citizens to represent selected roles and groups: a. Are they likely to authentically represent their role or group? b. Do they represent the axes of difference selected to be included in priority-setting? c. Do they see themselves as representing these axes of difference? d. Who selects them? • Members of their group or community • Local leaders/authorities • Organisers of the priority-setting process • Experts (researchers) • Other ____________________________ 2. Particular organisations to represent selected roles and groups: a. Do organisations exist that authentically represent the selected roles and disadvantaged groups? b. Do their memberships encompass the selected axes of difference? c. Do power disparities exist between selected organisations? d. Who selects individuals to represent organisations in priority-setting? • Their members • Their leaders • Organisers of the priority-setting process • Experts (researchers) • Other ____________________________	Which organisations or individuals will represent the selected roles and groups? Points to consider: 1. Does it make sense to ask community leaders, community members, or key informants to select individuals or organisations to represent the identified roles and groups? If yes, consider giving them some criteria that you’re hoping representatives will meet in order to avoid selection biases. 2. Do you have evidence that these organisations’ memberships reflect the group or roles’ diversity and are regularly consulted about their needs and priorities? 3. Where individuals will represent a role or group, do they collectively reflect its diversity and share lived experience with those they are representing? 4. Do any of the selected representatives have substantial financial conflicts of interest that you think will bias their identification of research priorities?
Framing	Is priority-setting framed in a non-neutral way that excludes some of their key health needs?	Will you make it clear to participants that any or most health research topics can be raised during priority-setting? OR Will it been made clear to participants that *not all* health research topics can be raised during health research priority-setting and why that is?
Having voice	1. In what ways will power suppress citizens’ agency in deliberations during priority-setting? 2. Will strategies be implemented to counteract the impact of these power dynamics on citizens’ agency in the deliberative process? 3. Will research priorities and questions be internally synthesised? By whom? • Citizens, including less powerful citizens • Researchers and citizens, including members of disadvantaged groups and less powerful citizens • Researchers and citizens • Researchers 4. If they’ll be externally synthesised, what is the justification? 5. Has local knowledge from citizens along axes of powerlessness and disadvantaged groups been used? If yes, which ones and why? If not, why not?	Facilitation 1a. Will you have a locally-based person facilitate focus groups or deliberations during priority-setting? If not, what are your reasons? 1b. How will the facilitation method/approach help equalise power dynamics between community members? Documentation 2a. Will you have a locally-based person document the priority-setting process? If not, what are your reasons? 2b. How will community members be given an opportunity to review the documentation of the priority-setting process? Synthesis 3. Will you give the voices of community members, especially those considered disadvantaged or marginalised, equal or greater weight than other voices when setting research priorities? If not, what are your reasons?
Leadership	1. Will the engagement process be initiated by: a. Local researchers and citizens, or citizens alone b. Foreign researchers, local researchers, and citizens c. Foreign researchers and local researchers d. Foreign researchers 2. If solely foreign researchers, what is the justification?	Who will initiate and lead engagement with community members during health research priority-setting?
Mass	1. Does the number of participants representing those who lack power over health decision-making balance or exceed the number of participants representing those who typically have such power?	1. Will the number of representatives of lower status community roles be equal to or exceed the number of representatives of higher status community roles at each stage of the priority-setting process? If not, what are your reasons? 2. Will a sufficient number of representatives of lower status roles be engaged in each stage of the priority-setting process? If not, what are your reasons?
Level of participation	1. Will decision-making be limited to (foreign) researchers, i.e. “experts”? 2. At what level will each group participate? a. Lay control b. Decision-making c. Consultation	1. Will you involve community members as collaborators (decision-makers) in priority-setting? If yes, in what stages of the priority-setting process? If not, what are your reasons? 2. Is it fair to bring these community members into the same decision-making space?
Stage of participation	1. Will those who typically have power over health decision-making—(foreign) researchers, policymakers—enter the process earlier than those who do not? 2. When will each group be included? a. Planning the process, b. Research topic solicitation and prioritization, c. Formulating the research question, and/or d. Designing the intervention	1. Will you involve community partners and members from the start of the priority-setting process? If not, what are your reasons? 2. Will less influential and lower status community roles be involve later and in fewer stages of the priority-setting process than higher status and more influential roles?

In relation to framing, interviewees identified the presence or absence of an “open scope” (i.e. no or very few topics related to health being off the table) as a key framing issue in research priority-setting practice. Open framing was described as a way to share power with communities. Where factors prevented having a completely open scope, sharing power meant being transparent about those constraints *before* starting priority-setting [24]. In terms of representation, interviewees clarified the definition of genuine representation in research. They also suggested that power-sharing can be facilitated where community leaders, community members, or key informants are asked to select representatives to participate in priority-setting but indicated there are pitfalls associated with doing so in some cases [25]. Ethical considerations related to framing and representation were revised to reflect these insights (Table 2).

Second, where interviewees identified additional ethical considerations in relation to the seven sites of power, they were incorporated. Considerations related to conflicts of interest were added under ‘representation’ and considerations related to documentation were added under ‘having voice’ (Table 2). Third, where there was largely congruence between ethical considerations identified through the published conceptual analysis and interviewees’ insights, minor changes to considerations’ wording were made (Table 2). These changes often related to phrasing questions in a simpler, more reflective way, which was suggested by interviewees as necessary to make the toolkit a robust project planning aid. Ultimately then, the empirical findings supplemented the prior conceptual work in regards to the seven sites of power described by both. They did not contradict or oppose the findings of the conceptual work.

### Implications for the ethical toolkit

Ethical considerations relating to the context and aftermath themes were added into the toolkit. Ethical considerations relating to additional sites of power under the process theme were also added. In the revised toolkit, refined ethical considerations for those sites of power identified by both previously published conceptual and empirical analyses replaced their corresponding considerations in the initial version of the toolkit.

## Empirical component results

### Suggestions for improving the ethical toolkit

Thematic analysis of interviewees’ suggestions for improving the preliminary ethical toolkit identified two main themes: content and language. Under content, interviewees’ comments related to the role of the toolkit, the assumptions underlying it, gaps in the toolkit, and its scoring system. Under language, interviewees’ comments related to revising certain terms used in the toolkit and simplifying technical language.

#### Role of the framework

Interviewees highlighted that the ethical toolkit could have either a *developmental* or *evaluative* role and that it was unclear, based on how its questions were phrased, which it was meant to have:

*So is it designed as a systematic review tool almost, something that you’d use to assess quality of completed research? Is it a developmental thing that you’d give to people to say think about these things when you’re doing this? I think some of your questions are geared towards helping people to do it better, other questions are clearly kind of implying well this is already completed, did they do this, and now I’m gonna score them*. (Ethicist)

If used prior to engaging community members in global health research priority-setting, the toolkit could comprise a formative exercise that prompts reflective discussions amongst research team members and helps them better design their priority-setting processes. This would require articulating toolkit questions in an open-ended way that leads people to think about and talk about their answers as a team. Researchers and community partners could write their collective answers to toolkit questions in free text boxes rather than ticking off items on a checklist. According to another ethicist, “if you have a checklist, it’s very prescriptive and then you know people would just tick boxes without really thinking about what they mean.”

If used after priority-setting, the toolkit could comprise an evaluation tool for assessing the quality of engagement with community members. It could be used by the research team and/or by community members. Involving community members in evaluation would be consistent with sharing power in accountability. Interviewees suggested the toolkit be redeveloped as either a developmental tool or an evaluative one and its purpose (as one or the other) be made explicit. They did not give their opinion on which of the two functions the toolkit should serve.

#### Toolkit assumptions

Interviewees drew attention to the fact that several assumptions underlay the initial ethical toolkit’s content. The toolkit takes a particular normative orientation—advancing social justice—as its starting point. To advance social justice, global health research priority-setting should help make marginalised communities’ health concerns and knowledge visible in research topics and questions. Priority-setting should also meaningfully engage them, which (in part) entails structuring processes in ways that challenge or minimise the impact of power dynamics between researchers and communities and between community members. The toolkit, therefore, assumes that disrupting existing power dynamics is appropriate and desirable and its questions seek to generate such effects. Yet this underlying normative orientation is open to challenge. Interviewees and focus group members queried whether, in adopting such a starting point, the toolkit might impose an overarching worldview and values on the communities where global health research is being performed. They suggested that the toolkit’s underlying normative views be clearly stated to users.

An interviewee who performs Indigenous health research in Australia (but was non-Indigenous herself) further noted that the toolkit’s content reflected a Western epistemology and thus may or may not be appropriate for use in Indigenous health research. S/he highlighted concepts used in the initial toolkit such as citizen, equal voice, and priority-setting as being Western ideas, making the following observations:

*So even the idea of talking about citizens, so citizens, so you’d have to find a way to use different words… if you replace the word citizen with community, citizens are individuals communities are collectives….*

*One of the things that you have to get used to is that in some cultural contexts particular voices count, and it’s not a matter of a Western view of everyone gets the same say. So in more traditional Aboriginal communities elders voices count and elder voices will count even if what you’re talking about is something to do with young people…*

*You see Aboriginal researchers doing priority setting, and Aboriginal communities doing priority setting, but I don’t know whether that’s a thing in an Indigenous epistemology, whether it makes sense to say well we’ve gotta put these things in order of ones’ better or more important than others.*

S/he also stated that working with Indigenous people to disrupt power imbalances as they relate to relationships between Indigenous people and non-Indigenous people could work, but that efforts to disrupt power imbalances *within* Indigenous communities, particularly when carried out by non-Indigenous individuals or “outsiders”, would likely be problematic. Thus, the toolkit will need further adaptation and piloting before it can potentially be used to design priority-setting processes for Indigenous health research projects.

#### Gaps

In accordance with previously described results, interviewees identified two gaps in the toolkit’s content as being questions related to context and accountability. For example, an interviewee said there are alternative pathways for learning from marginalised communities beyond formal priority-setting, particularly in contexts where researchers have been embedded in communities over a long period of time. S/he emphasised that such alternative pathways should not be discounted or ignored in favour of new priority-setting processes, which may be very resource intensive.

It was also noted that the toolkit lacked an *asset-based* orientation and, instead, exhibited a deficit-based approach. Rather than thinking about relying on and strengthening the assets that communities have, the initial toolkit reflected a view of communities as having deficits that need to be overcome. A researcher affirmed the importance of building an asset-based approach into the toolkit:

*The point is when people, communities start thinking about themselves as having the capacity to create and get information and knowledge based on their experience, rather than victims in a process, it also really changes the, the dynamic. So I think it’d be really interesting to think about in building the framework is, have you tried to identify assets of the community and does the process reflect the potential assets rather than compensate for the deficits*.

Providing users of the toolkit with an explanatory document was identified as essential by interviewees. They made numerous suggestions for what information to include in that document. Finally, interviewees recommended that the toolkit offer practical guidance on what actions researchers and community partners can take based on their answers to toolkit questions.

#### Scoring system

Interviewees felt that the scoring system employed by the preliminary toolkit served to unblind the exercise. Ticking yes to its questions was associated with being ideal, ethically preferable, minimally ethically acceptable, or ethically problematic. Essentially, it alerted users to the fact that one’s answer to each question was morally good or bad, which might lead them to tick yes only where it was considered to be ideal or ethically preferable. Having users score their answers after answering all toolkit questions was recommended. Having an explanation of why answers were ideal or ethically preferable versus minimally ethically acceptable or ethically problematic was also said to be important to include in the toolkit. Without such an explanation, it was unclear why certain answers were better than others and could appear arbitrary. An interviewee stated that s/he found the scoring system to be “judgmental”.

#### Problems of language

The initial toolkit used the term ‘citizen engagement’ but the term ‘citizen’, which is drawn from the development studies literature,^5^ was not familiar to many interviewees:

*I was thinking of citizens as non-experts and as health workers as part of the expert power holders usually… I think then you might need to be careful with quite a lot of the wording of citizens… make it more similar to the kind of wording that’s used in health policy and systems research to sort of health workers, at the frontline, sort of senior policy makers, that kind of thing and then more the general public and particularly disadvantaged members of the general public, that kind of thing… Why are you using the word citizens? I’ve never heard it.* (Researcher)

Using the term ‘community’ rather than ‘citizen’ was suggested as another option.

The initial toolkit’s use of the binary foreign researcher and local researcher distinction was cited as problematic by interviewees. They noted that the category of local researcher can comprise several types of differently positioned actors. As an example, a researcher said

*if I looked at the people at the medical school who are the research partners, they’re very powerful and what they represent is very powerful in comparison with the community that they were targeting. Whereas in Nicaragua because it was an NGO that was doing community research and they, and the people that they hired to do research were like people who were basically had a background in like community work and were much more like already engaging in those communities. Both could be technically qualified as local, right*.

To better reflect this complexity, interviewees suggested distinguishing between “national” and “locally-based” researchers. Locally-based researchers refer to individuals who are in a continuous relationship where they are known and trusted by the community. They typically share its culture and have lived in or nearby the community for many years. This was contrasted with national researchers who “parachute in” from the capital city or other parts of the country.

A common critique of the initial toolkit was its use of overly complicated, technical language. The toolkit is intended to be used by researchers, whose first language may not be English, and community partners (e.g. service providers, policymakers, COs). Interviewees recommended simplifying the terminology used in the toolkit and pilot testing the toolkit with low and middle-income country community advisory boards, COs, and low and middle-income country ethics review committees.

### Implications for the toolkit

The toolkit’s potential to serve as a developmental or evaluative tool was taken under consideration and it has been revised to better function as a *developmental* instrument. Helping researchers and their partners improve priority-setting processes’ design comes prior to evaluating priority-setting processes. As such, it was thought most helpful to maintain the toolkit as a developmental instrument but to strengthen its capacity to generate reflective discussions. Interviewees said a toolkit that prompts reflective discussions comprises a robust developmental instrument for designing priority-setting processes. All ethical considerations in the toolkit have thus been phrased or re-phrased as open-ended questions for discussion. (Please note, this is already reflected in how the new and refined ethical considerations were phrased in the Conceptual Component Results section.) The revised toolkit does not contain a scoring system. At a later stage, a second version of the toolkit for use in evaluating priority-setting processes may be developed.

A companion document was written and added to the toolkit (Additional File 1). That document explains why the toolkit is needed, what its purpose is, what assumptions and values underpin the toolkit, how it was developed, who should use it, and how it should be used. The Companion Document also provides users with guidance on how to understand the questions posed by the toolkit worksheets and why they are important. To further incorporate an asset-based approach into the toolkit, it now includes several ethical considerations in relation to the site of power: community assets. Additionally, the toolkit provides researchers and their partners with suggested Next Steps to take after answering toolkit questions. For certain questions, these Next Steps call for working with key informants from the community to define and undertake tasks.

In relation to addressing problems of terminology, the toolkit has been revised to use the term ‘community’ rather than ‘citizen’ to achieve consistency with the engagement language used in global health research. The term community is defined in the Companion Document. The toolkit has been revised to use the terms national and locally-based researchers, which are also defined in the Companion Document. Technical terms identified by interviewees as difficult to understand (e.g. axes of difference, internal synthesis, external synthesis, means, ends, agency) have been removed from the toolkit. At this point, the toolkit has not been adapted for use in Indigenous health research priority-setting.

## The ‘improved’ toolkit

The initial version of the toolkit was a single checklist document focused on ethical considerations relevant to power-sharing *during* global health research priority-setting. It was missing considerations related to the context in which the priority-setting process occurs and what happens after the process ends, which are both key components of priority-setting. The initial toolkit sought to promote improvements to priority-setting processes’ design by helping researchers and their partners identify ethically problematic aspects of proposed processes. It did not include suggestions or strategies for sharing power where the toolkit identified problems with how a given priority-setting process was structured. The toolkit lacked explanatory materials and, as a result, it was unclear whether it was intended as a developmental or evaluative instrument, who was to use it, and when.

The toolkit remains a developmental tool and is now comprised of three worksheets—Worksheet 1: Selecting a CO Partner; Worksheet 2: Deciding to Engage, and Worksheet 3: Designing Priority-setting—and a Companion Document (Additional Files 1, 2, 3, 4, 5 and 6), rather than a single checklist. The reflective questions in Worksheets 1 and 2 correspond to the ethical considerations identified under the context theme. The reflective questions in Worksheet 3 correspond to the ethical considerations identified under the process and aftermath themes. The worksheets are to be completed by researchers and their *community* partners (e.g. health care providers, policymakers, community organisations) *as a team* before commencing priority-setting for a given global health research project. The Companion Document and worksheets provide ideas and example strategies for sharing power with communities in global health research priority-setting. These are drawn from the key informant interview data [24, 25].

When using the toolkit, the first step is for researchers and their partners to read the Companion Document. Next, completing Worksheet 1 together will help them think about and collectively determine whether the research team can be strengthened by adding a (or an additional) CO partner. Once the research team is finalised, Worksheet 2 helps its members determine whether engagement is necessary and can be meaningfully done with a given community.

Where engagement is necessary and sharing power with the given community is possible, Worksheet 3 then helps research teams design the priority-setting process for a given global health research project. Its questions are grouped by sites of power during and after health research priority-setting. They correspond to the key ethical considerations relating to all sites of power identified through the published conceptual and empirical work.

## Future directions

The revised toolkit is more comprehensive and robust than the initial version. Yet further work can continue to strengthen it. Both the conceptual work canvassing and applying concepts from literature on participation [23] and the empirical work gathering information from practice [24, 25] had limitations. While saturation was seemingly achieved in the previously published conceptual work, it has since been pointed out that it missed literature describing concepts identified by interviewees. Thus, it will be important to continue identifying and analysing existing literature on participation to further inform the toolkit.

Although the empirical work initially sought to interview community members who had been or are involved in health research priority-setting, recruiting them proved extremely challenging. This was largely due to how uncommon it is for community members to be involved in decision-making about health research topics and questions. To obtain a more community-based perspective, CO staff were interviewed. While saturation was achieved in this study, community members are, nonetheless, a key perspective to obtain when exploring how to share power with them during health research priority-setting. Future case study research performed as part of this research program will focus on capturing their perspective and incorporating it into the toolkit. Second, a large proportion of interviewees came from three regions: UK and Europe, Australia, and Africa. Future research could better acquire perspectives from interviewees from Latin America, South America, the Eastern Mediterranean region, and Asia.

The ethical toolkit is thus still a work in progress. Next steps include analysing and applying concepts from additional literature on participation, pilot testing the toolkit, and undertaking interviews with community researchers and consumers involved in research as well as the aforementioned case study research. The latter will ensure the voices and perspectives of community members are better reflected in the toolkit’s content.

## Conclusions

The process described in this paper significantly strengthened the ethical toolkit. Toolkit questions are now more comprehensive, spanning all phases of priority-setting (prior to, during, and after) and more sites of power. They are open-ended and worded to prompt discussion and help research teams design better priority-setting processes. Where global health researchers and their partners reflect on and discuss the questions in Worksheets 1, 2, and 3 and then finalise their priority-setting processes’ design, it will create more inclusive processes that are less likely to reinforce hierarchies of privilege and subordination. This, in turn, will help deliver projects with research topics and questions that more accurately reflect the health care and system needs of marginalised communities.

## Supplementary information

**Additional file 1.** Sharing Power with Communities in Priority-Setting for Health Research Projects: A Toolkit. Companion Document.

**Additional file 2.** Sharing Power with Communities in Priority-Setting for Health Research Projects: A Toolkit. Worksheet 1.

**Additional file 3.** Sharing Power with Communities in Priority-Setting for Health Research Projects: A Toolkit. Worksheet 2.

**Additional file 4.** Sharing Power with Communities in Priority-Setting for Health Research Projects: A Toolkit. Worksheet 3A.

**Additional file 5.** Sharing Power with Communities in Priority-Setting for Health Research Projects: A Toolkit. Worksheet 3B.

**Additional file 6.** Sharing Power with Communities in Priority-Setting for Health Research Projects: A Toolkit. Worksheet 3 Supplemental Table.

## Data Availability

The de-identified datasets used and/or analysed during the current study are available from the corresponding author on reasonable request.

## Abbreviations

CBO: Community-based organisation
CIOMS: Council for International Organizations of Medical Sciences
NIAID: National Institute of Allergy and Infectious Diseases (US National Institutes of Health)
UNAID: Joint United Nations Programme on HIV/AIDS

## Footnotes

1 By adopting a reflective equilibrium approach, this research program took a consultative orientation. Doing so enabled accessing the insights of key informants who work across multiple disciplines and in numerous countries. Having a consultative orientation meant an external thinker (BP) gathered the data, analysed it, and developed the study’s conclusions [9].

2 Questions posed by toolkit worksheets may be used to inform engagement in other phases of research projects. However, it is beyond the scope of this paper to adapt them for use beyond the priority-setting phase. The toolkit’s focus on engagement in priority-setting is not meant to imply it is sufficient to include communities in that phase of research alone. Community engagement is critical in all phases of research projects [6, 28].

3 The study initially sought to interview community members who had been or are involved in health research priority-setting, but recruiting such individuals proved extremely challenging. This was largely due to how uncommon it is for community members to be involved in decision-making about health research topics and questions, which was a key theme expressed by numerous interviewees in this study. To obtain a more community-based perspective, CBO staff were ultimately interviewed.

4 This section summarises some of the empirical data on components of engagement and sites of power that is reported in Pratt [24]. The data is summarised to make clear how the ethical considerations characterised in this paper were identified. Pratt [24] does not compare the empirically-identified components and sites of power to the prior conceptual work or derive ethical considerations related to additional components and sites of power.

5 The citizen label recasts lay participants as agents, with the right to shape and define decision-making spaces, set agendas, and bring their local systems of knowledge to the for (Visvanathan 2005; Gaventa 2004; Cornwall 2004).
