# Supplementary material for: Developing a toolkit for engagement practice: sharing power with communities in priority-setting for global health research projects
Source: BMC Med Ethics. 2020 Mar 14;21:21. doi: 10.1186/s12910-020-0462-y (PMC7071780; doi:10.1186/s12910-020-0462-y)
Supplement: Supplementary file 1 — Additional file 1. Sharing Power with Communities in Priority-Setting for Health Research Projects: A Toolkit. Companion Document. [file 12910_2020_462_MOESM1_ESM.docx]

**Sharing Power with Communities in Priority-Setting for Health Research Projects: A Toolkit**

**Dr Bridget Pratt**

**School of Population and Global Health**

**University of Melbourne**

**Companion Document for Worksheets 1-3**

Preamble

1. Why is the toolkit needed
2. What is the purpose of the toolkit
3. What assumptions and values underpin the toolkit
4. How was the toolkit developed
5. What is the toolkit
6. Who should use the toolkit
7. How should the toolkit be used

Definitions

Key Considerations in Worksheet 1

1. Partners

Key Considerations in Worksheet 2

1. New versus existing relationship
2. Existing priorities
3. Foundations of meaningful engagement

Key Considerations in Worksheet 3

1. Leadership

2. Empowerment

3. Diversity within the community

4. Stage of participation

5. Level of participation

6. Representation

7. Mass

8. Community assets

9. Space

10. Framing

11. Ground Rules

12. Facilitation

13. Documentation

14. Synthesis

15. Resources and compensation

16. Unintended harms

17. Respect

18. Accountability

**Preamble**

**Why is the toolkit needed?**

Helping improve health care and systems for those considered disadvantaged and marginalised is an important goal motivating the conduct of health research worldwide. Their *engagement* in priority-setting is a key means for setting research topics and questions of relevance and benefit to them.

But communities, especially those considered disadvantaged and marginalised, rarely have a say in the agendas and priorities of the very health research projects that aim to help them.

And even where their engagement occurs, without attention to power dynamics, it can lead to presence without voice and voice without influence. Voices are excluded from priority-setting, particularly those already disadvantaged or marginalised by their societies’ institutions and norms. Existing evidence confirms that, for example, being *female*, being *poor*, having *little formal education*, living with a *disability*, and/or belonging to certain *ethnic groups* means community members are listened to less or not at all in health priority-setting.

Where voices from marginalised communities aren’t heard, health research projects won’t prioritise the key problems they face in accessing and affording health care and services. Without their input, projects are much less likely to generate evidence that will improve health care and systems for them.

It is, therefore, very important to carefully design health research priority-setting processes to share power with communities.

**What is the purpose of the toolkit?**

The toolkit aims to help researchers and community partners design priority-setting processes that will make the health needs and knowledge of communities, particularly those considered disadvantaged and marginalised, more visible in health research projects’ topics and questions. The toolkit is a reflective project planning aid for use *before* priority-setting is undertaken for a health research project.

**What assumptions and values underpin the toolkit?**

Underpinning the toolkit is a view about the goal of community engagement in health research priority-setting—namely, that it should promote values of *social justice* and *equity*. Two core aspects of social justice are addressing the health needs of those considered disadvantaged and marginalised and supporting their participation in decision-making. To promote social justice and equity, priority-setting should help make the health concerns and knowledge of such communities visible in health research topics and questions. Priority-setting should also involve their meaningful engagement and participation. This means community members are able to have a say and influence the priority-setting process and its outputs.

A second core assumption of the toolkit is that researchers will typically work with community partners (e.g. health care providers, policymakers, community organisations) to conduct projects and engage with the wider community (i.e. community members who are not part of the research team) when setting those projects’ research topics and questions. In some cases, researchers may initiate the partnership with community partners. In other cases, community partners may initiate the partnership with researchers. The toolkit assumes that both researchers and community partners will then initiate the engagement process with community members to set projects’ research topics and questions.

A third core assumption of the toolkit is that, while its focus is on priority-setting, community engagement is also required in other phases of the research process: intervention design, data collection and analysis, and dissemination. Their engagement in priority-setting alone is necessary but not sufficient. Toolkit questions, however, have been designed to inform health research *priority-setting*. They *may* apply to community engagement in other phases of research but have not been written with them in mind.

A fourth core assumption of the toolkit is its definition of community. The toolkit takes a broad definition of community. Communities can be based on geography; on shared interests or goals; or on shared characteristics, situations or experiences, including experiences of marginalisation. They encompass (amongst others) community leaders and elders, non-aligned community members (the general public), and people who are part of the health system in that community: namely, patients, health care providers, health care managers, insurers, policymakers, and others.

**How was the toolkit developed?**

Development of the toolkit occurred over a three-year period and was led by a bioethicist as part of an Australian Research Council Discovery Early Career Researcher Award. The questions presented in toolkit worksheets were identified based on both conceptual and empirical ethics research. First, six key bodies of literature were analysed for sites of power related to participation. They include development literature, political philosophy literature, ethics literature, health priority-setting literature, public deliberation literature, and community-based participatory research literature. Conceptual work was then undertaken to identify ethical considerations related to power-sharing at each site in health research priority-setting. This generated an initial version of the Designing Priority-setting Worksheet.

Sites of power and ethical considerations identified by the conceptual work were then tested against and informed by the experiences and perspectives of researchers, ethicists, community engagement practitioners, and community-based organisation staff. Those recruited for interview came primarily from Australia, Europe, and Africa and, to a lesser extent, from North America, Latin America, and Southeast Asia. Based on that empirical work, the Designing Priority-setting Worksheet was revised and the Selecting Partners, Deciding to Engage Worksheet, and Companion Document were developed.

**What is the toolkit?**

The toolkit for ***Sharing Power with Communities in Priority-Setting for Health Research Projects*** is a set of three worksheets and a companion document.

*Worksheet 1* helps research teams think about and collectively determine whether they can be strengthened by adding a (or an additional) research institution or community partner.

Once the research team is finalised, *Worksheet 2* helps its members reflect on and collectively determine whether engagement is necessary in priority-setting and, if so, whether it can be meaningfully done with members of a given community.

Where meaningful engagement is necessary and possible, *Worksheet 3* then helps research teams design the priority-setting process for a given health research project. Reflecting on and collectively answering Worksheet 3 questions will promote the design of priority-setting processes where power is more evenly shared with communities, particularly those considered disadvantaged and marginalised.

The *Companion Document* provides toolkit users with guidance on how to understand worksheet questions and why they are important.

**Who should use the toolkit?**

Researchers and their community partners (e.g. health care providers, policymakers, community organisations) should use the toolkit together. This means completing Worksheets 1, 2, and 3 as a team.

Given the toolkit’s underlying values and assumptions, it may be especially suited for (but not limited to) use in health research projects that aim to advance health equity and social justice as well as in community-based health research projects.

**How should the toolkit be used?**

The toolkit is a reflective project planning aid for use *before* undertaking priority-setting for a health research project. It should be used to develop and inform a final priority-setting plan as follows:

**
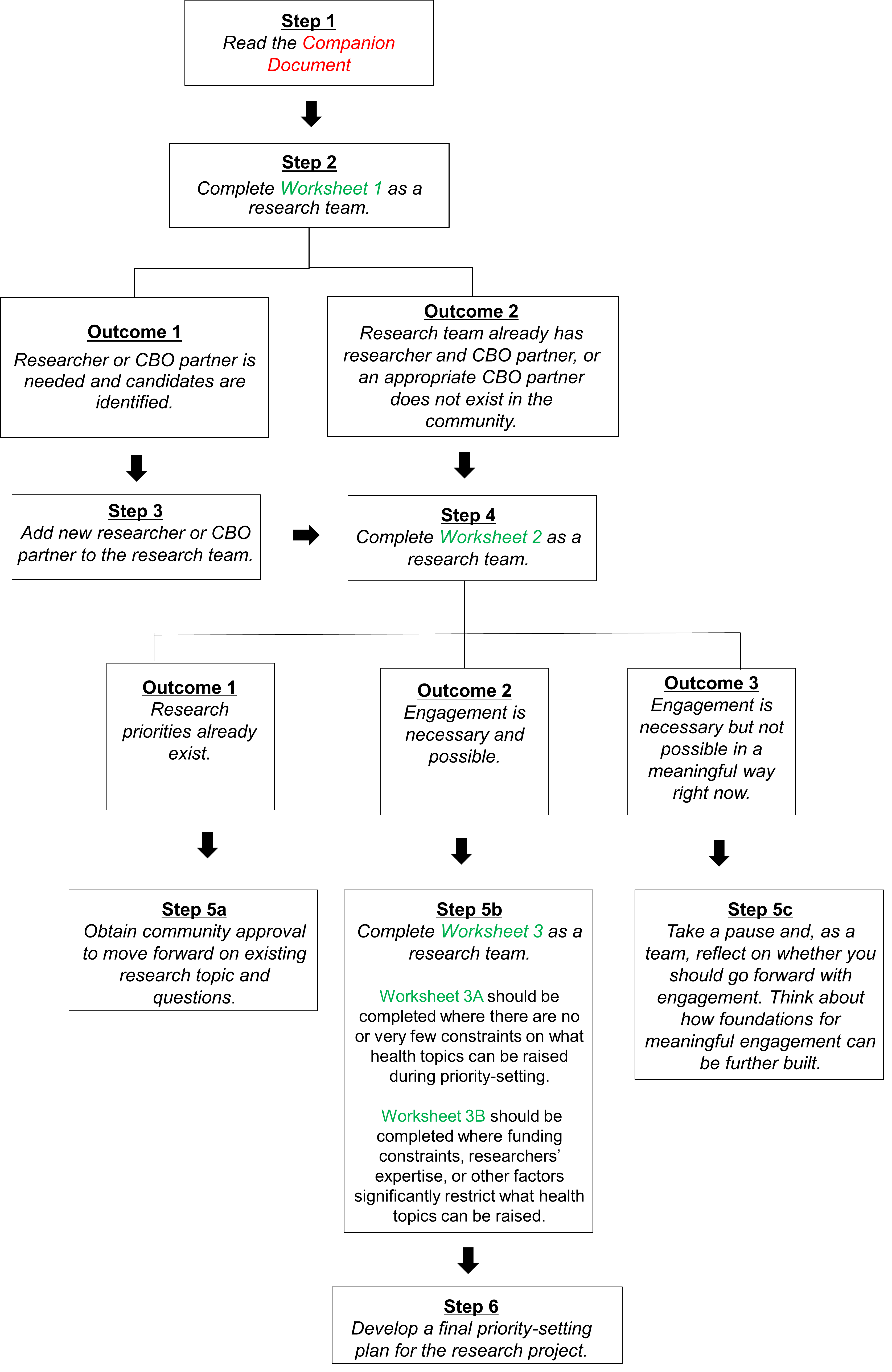
**

**Definitions**

**Community assets** = Resources, information, and skills within the community that can be used to strengthen health research priority-setting processes.

**Community members** = Community membership can be based on geography; on shared interests or goals; or on shared characteristics, situations or experiences, including experiences of marginalisation. Communities encompass (amongst others) community leaders and elders, non-aligned community members (the general public), and people who are part of the health system in the given community: namely, patients, health care providers, health care managers, insurers, policymakers, and others. Community members are not part of the research team that initiates the priority-setting process.

**Community partner** = Community-based organisations, health care providers, and/or policymakers that partner with researchers to conduct health research projects. They are part of the research team and are involved as decision-makers in most or all stages of the research project.

**Conflict of interest** = Having a substantial financial interest in the outcome of priority-setting, e.g. patient organisations funded by pharmaceutical companies.

**Consultant** = Individuals are invited to give their input and to provide information but have no assurance that it will be used by decision-makers to set the health research topic or questions for a given project.

**Decision-maker** = Individuals are part of the group that has the final say on what research topics and questions are selected for a given project.

**Empower** = Building individuals’ knowledge, confidence, relationships, identities, and capacities and/or altering the social relations and institutions that shape individuals’ capacities and actions.

**Engagement** = A process of inclusive participation and working collaboratively with community members in health research priority-setting.

**Health research priority-setting** = Setting the research topic and formulating the research objectives and questions for a health research project.

**International researcher** = Researchers from universities or other organisations from outside the country where the health research project is being conducted. They are often from high-income country universities or organisations.

**Locally-based** = Individuals who are in a continuous relationship where they are known to, and trusted by, the community. They typically share its culture and have lived in or nearby the community for many years.

**National researcher** = Researchers who are from the country where the health research project is being conducted but are not locally-based in the community where it is being done.

**Reflective knowledge** = Knowledge based on prior engagement with a topic and reflection on it. Contrasted with knowledge based on opinion, belief, or received wisdom. Developing reflective knowledge requires prior opportunity to engage with information about a topic, to ask questions, and to take time to think about it and to come back to it.

**Stepped approach** = Facilitation method where small groups with some degree of homogeneity or similar characteristics meet first to deliberate on a topic (e.g. about what research priorities to set). Then those small groups come together as a large group to deliberate about that topic.

**Key Considerations in Worksheet 1:**

**Selecting Partners**

**1. partners**

**Does your research team include a research partner(s) and a community partner(s) who can access a community that is considered disadvantaged and/or marginalised?**

Why this consideration is important

Working with research partners can enable community partners like health care providers, policymakers, community-based organisations (CBOs) to conduct research that is needed by the communities they serve. It is important to select research partners who share community partners’ values and have expertise in the kinds of research that can benefit the community.

Working with community partners can facilitate power-sharing with those considered marginalised and disadvantaged. When working with CBOs as community partners, this often happens where CBOs have certain values, capacities, and social standing in their community. Thus it is important to select CBO partners that:

- have social justice and equity as guiding values,
- have leaders, staff, and members that reflect the community’s diversity,
- have leaders, staff, and members that live in the community,
- have peer-led community groups,
- have strong networks within their community,
- do grassroots work and outreach with those considered to be disadvantaged and marginalised, and/or
- have non-elite status.

As an example, working with a local NGO in comparison to a medical school can make a difference in terms of accessing the voices of those considered to be disadvantaged or marginalised. Medical school staff can often be seen as powerful elites, which may make members of such groups less likely to speak up in front of them. In contrast, NGO staff can often have a reputation for being more community-based and have experience working with these groups.

When working with health care providers or policymakers, it is important to select partners who can access those considered disadvantaged or marginalised *within the health system*, e.g. community health workers or patients with certain stigmatised illnesses.

**Key Considerations in Worksheet 2:**

**Deciding to Engage**

**1. New versus existing relationships**

**Have you engaged in research or other work with this community before?**

Why this consideration is important

A distinction is made between long-term and oasis engagement, while acknowledging a spectrum exists between them. Oasis engagement is defined as the research team initiating a brand new set of relationships and opening a dialogue with a community as part of a single project. Long-term engagement or embeddedness means researchers and/or their community partners have worked and spent time in the same setting for many years, building relationships. They will have conducted numerous projects where community members’ ideas and views were sought. Such projects may be research projects, health programs, or community development projects. The foundations for meaningful engagement are much *less* likely to be present in contexts of oasis engagement or where members of the research team have not worked with a given community for very long.

**2. existing priorities**

**Have the community’s health research priorities, including those of the less well-off, less influential, and/or marginalised, already been voiced?**

Why this consideration is important

Where researchers and community partners have been embedded in a given community for a long period of time, it is likely that previous studies have been done where community members’ views and ideas, including those of the less well-off, less influential, and/or marginalised, have been sought and speak to or explicitly articulate their health research priorities. Where community health research priorities have already been articulated and documented, then it may not be necessary to undertake engagement activities in priority-setting for new projects. Doing so, in fact, might comprise a poor use of resources that could be better spent on other research activities or engagement in other phases of the research process (e.g. data collection, data analysis, dissemination).

**3. foundations for meaningful engagement**

**Do the foundations for sharing power in priority-setting exist with this community?**

- **Do the research team and community members have the capacities necessary for sharing power in health research priority-setting?**
- **Does the social, political, institutional, and funding context support sharing power in health research priority-setting?**

Why this consideration is important

The research team and community members need to have particular capacities for power sharing to occur in health research priority-setting. Community members need to understand research (what it is, what it can and can’t deliver, what questions it can and can’t answer), priority-setting processes, and how health systems function. They also need to have reflective knowledge on health topics based on prior engagement with the topics and reflection on them. Such knowledge is generated by having opportunity to engage with information about topics, to ask questions, and to take time to think about that information and to come back to it. It is necessary for community members to identify which topics are urgent to address through research. At the start of relationships between researchers and communities, community members may not have all these capacities. If this is the case, these capacities should be strengthened before priority-setting commences and/or built over the course of the priority-setting process (see Key Consideration: Empowerment). They can also be built through wider community engagement programs at the institutional level like that run by the Kenyan Medical Research Institute in Kilifi.

Members of the research team, in turn, need to be good listeners, open-minded and flexible to undertaking new research directions that emerge from engagement; and be sensitive and empathetic to poverty and disadvantage. They should not buy into hierarchies of knowledge that designate local ways of knowing as inferior to expert knowledge. They also need to understand community values, norms, power dynamics, and structures of representation and have the skills and training necessary to perform engagement. Some members of the research team may be relied on more than others for their capacity to understand the given community and perform engagement. These might be locally-based researchers, community engagement practitioners, or community partners. At a minimum, however, other members of the research team should try to increase their capacities in these areas during projects.

Broader social, political, institutional, and funding environment factors also affect whether power sharing is possible in health research priority-setting. For example, where communities have previously been exploited by researchers, this comprises a substantial barrier to meaningful engagement. The resultant lack of trust makes it especially difficult to engage community members, including those considered disadvantaged and marginalised, to participate in priority-setting. Significant time and relationship-building will likely be required to overcome such a barrier. This can be facilitated where researchers spend time in the community and/or help community partners outside of research projects. And through the community engagement programs of research institutions.

**Key Considerations in Worksheet 3:**

**Designing Priority-setting**

Priority-setting processes consist of 4 components: who initiates, for what purpose, who participates, and how they participate during the process. The considerations in Worksheet 3 relate to these different components of priority-setting, as depicted below (Figure 1). Three considerations are overarching and do not pertain to specific components: community assets, resources and compensation, and unintended harms.

**
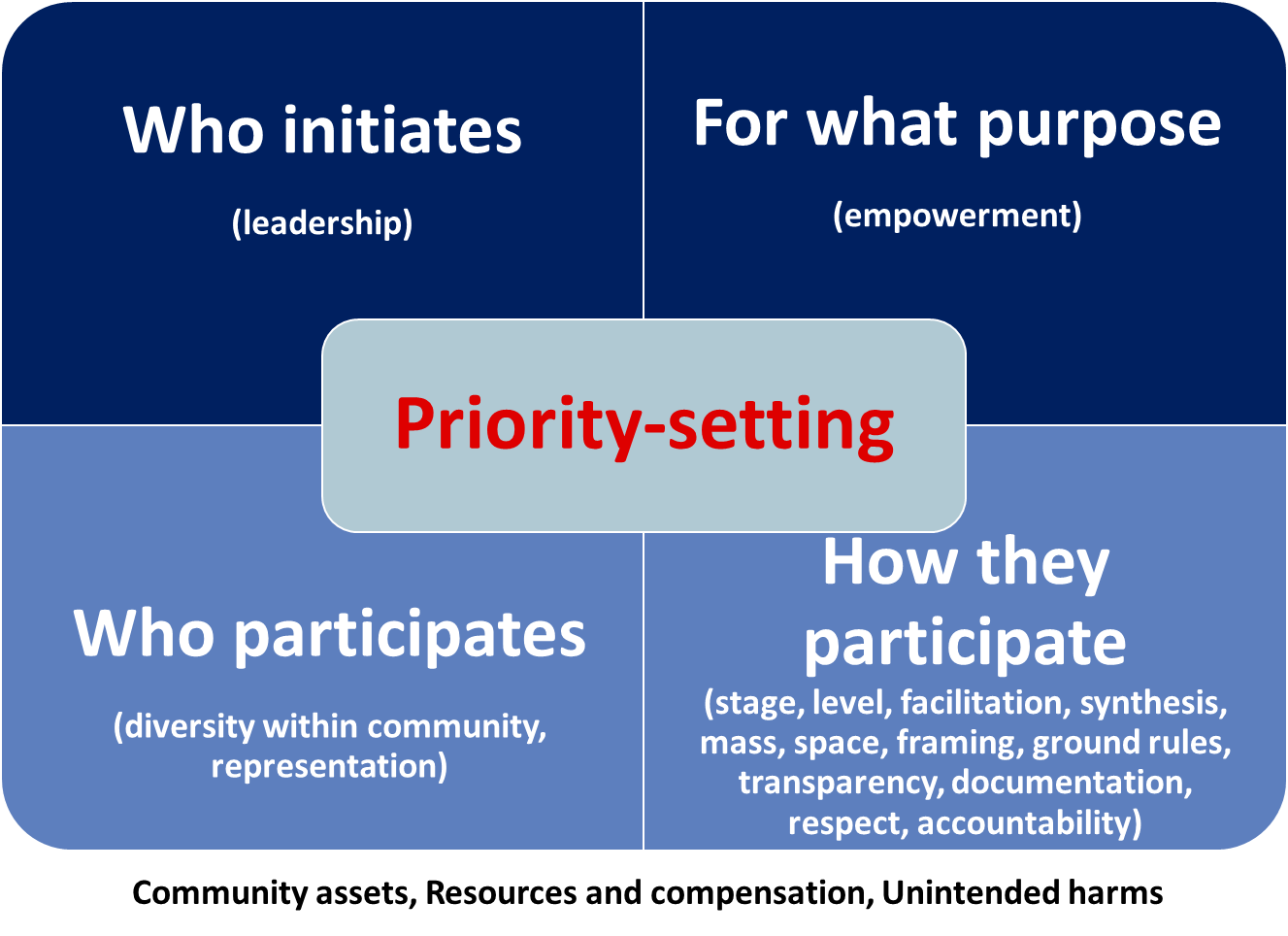
**

Figure 1: Components of priority-setting and their associated ethical considerations

**1. Leadership**

**Who will initiate and lead engagement with community members during health research priority-setting?**

Why this consideration is important

A distinction is made in the development studies literature between ‘invited spaces’ and ‘created spaces’. Invited spaces are those into which people are invited to participate by various kinds of power-holders or authorities. Created spaces are those that are claimed by the less powerful from the power-holders or created by them. They are often spaces that emerge organically out of common concerns or identifications experienced by marginalised groups in a society.

The implication of these concepts for engagement in health research priority-setting is, first, to recognize that, in practice, such engagement is typically an invited space, where international and national researchers invite locally-based researchers, community partners, and (more rarely) community members to participate. Two types of power dynamics are then often recreated within such spaces—namely, those of:

- ‘expert’ researchers over community members and
- international or national researchers, who “parachute” in from the capital city or a foreign university, over locally-based researchers and community partners.

To avoid reinforcing such power dynamics, it is important that locally-based researchers and community partners are amongst those initiating community engagement in health research priority-setting.

**2. EMPOWERMENT**

**Will community partners’ and members’ capacities be strengthened during priority-setting?**

Why this consideration is important

Two main types of goals exist for community engagement: instrumental and transformative. In health research, community engagement is often discussed as having an instrumental goal—namely, as being a means to enhance the relevance of research questions to communities and to promote research translation by generating buy-in from policymakers, healthcare providers and other key stakeholders. Less common are engagement processes that aim to empower community partners and members, e.g. build their knowledge, confidence, networks, and capacities to undertake priority-setting. These affective and cognitive changes can ultimately lead to behaviour changes that challenge unequal power relationships. For example, changes where a community or a community partner identify their own research priorities and then require all studies done with the community to align with those priorities.

Where engagement in research priority-setting only has instrumental purposes, this limits its capacity to reduce perceived inequalities in power between researchers and communities. It will fail to empower communities in ways that support their right to help set research agendas and is, therefore, not ideal.

**3. diversity within the community**

**3a. Which community roles will you engage during priority-setting and for what reasons?**

**3b. List which of the roles identified in Q3a correspond to greater or lesser influence and status within the community.**

**3c. Who are considered disadvantaged, less influential, lower status, or marginalised within these roles?**

**3d. Which of these groups or stakeholders will you engage and for what reasons?**

Why this consideration is important

Ideally, sharing power demands a *range* of community members are present or represented during health research priority-setting so that processes are informed by differently situated actors.

Achieving range means that participants span a wide spectrum of roles in a community *and* include those considered disadvantaged, less influential, lower status, or marginalised within the community. Pooling the knowledge of all social positions maximises the information base used to set health research priorities. Listening to others who speak from different, less privileged perspectives will also lead participants to transform their opinions on what research priorities should be from narrow expressions of self-interest to a more complete account of what research is needed to serve the population as a whole, especially the disadvantaged and marginalised.

Where the selected community is based on geography or on shared characteristics, relevant roles to include may be community leaders and community members. Within such communities, those considered less influential or less likely to have a voice might be women or those living in remote areas. Those considered disadvantaged might be individuals living in settlements with less land or animals. Those considered marginalised might be groups that experience stigmatisation and/or oppression. Where the selected community is based on being part of the health system, relevant roles to include may be patients, carers, health care providers, policymakers, insurers, health care managers, and others. Within the health system, those considered less influential might be local policymakers (compared to Ministry of Health staff). Those considered marginalised might be community health workers (compared to other types of health care providers) or patients with certain stigmatised illnesses.

OR

Community roles that might be relevant to include in health research priority-setting are:

- community leaders,
- non-aligned community members (the general public),
- patients,
- families and carers,
- health care providers,
- purchasers,
- health care managers, and
- policymakers.

Each role likely brings knowledge or experience that will inform the health research agenda. Their inclusion may also serve impact (their having the power to take the research agenda forward) and/or legitimacy (their presence is required for the outputs of health research priority-setting to have legitimacy). Roles of lower influence or status may often be non-aligned community members (the general public) and patients.

Some examples of who might be disadvantaged, less influential or marginalised *within* these roles are given below:

- Community leaders considered less influential or less likely to have a voice might be women or those living in remote areas.
- Policymakers considered less influential might be village-level policymakers (compared to district health staff).
- Community members considered to be disadvantaged might be individuals living in settlements with less land or animals, or that grow crops generating less income.
- Community members considered to be marginalised might be groups that experience stigmatisation and/or oppression.
- Health care providers and patients considered marginalised might be community health workers (compared to doctors and nurses) and patients with certain stigmatised illnesses.

**4. stage of participation**

**4a. Will you involve community partners and members from the start of the priority-setting process? If not, what are your reasons?**

**4b. Will lower status and less influential community roles be involve later and in fewer stages of the priority-setting process than higher status and more influential roles?**

Why this consideration is important

The stage in decision-making at which individuals begin to participate significant. Earlier entry is associated with higher quality participation. It means making a greater number of decisions, including those that determine how the decision-making process is organised. Individuals thus have a greater opportunity to raise their voice and be heard. Being part of more stages in the priority-setting process is also associated with higher quality participation.

In priority-setting for health research projects, a number of stages of participation can be identified:

- 1. planning the priority-setting process,
  2. research topic solicitation,
  3. research topic prioritization, and
  4. formulating the research objectives and question(s) and interventions.

Research topic solicitation means coming up with a list of possible health problems or needs upon which to focus the research project. In some instances, this may entail collecting and analysing data to generate a list of health problems or needs. Research topic prioritization means selecting a single health problem or need from that list to be the project’s focus. Research objectives, question(s), and/or actions to take (interventions) for the project are then developed that relate to the selected health problem or need. These objectives and questions reflect what knowledge needs to be collected about the health problem or need to better understand it and/or how to address it. Interventions are strategies or activities to address the health problem or need.

Stages 2-4 can be run as part of a single priority-setting exercise or separately. For example, a project might first undertake an exercise to identify research topics. And then run a second exercise to prioritise a topic and identify research questions to study under that topic.

The *start* of the priority-setting process comprises *planning the process* and/or *research topic solicitation*. Reducing perceived power inequalities would support the early entry of locally-based researchers, community partners, and community members, including those with roles that are considered disadvantaged, less influential, and/or marginalised. It would also support their being involved during all or at least several phases of the priority-setting process.

**5. level of participation**

**5a. Will you involve community members as collaborators (decision-makers) in priority-setting? If yes, in what stages of the priority-setting process? If not, what are your reasons?**

**5b. Is it fair to bring these community members into the same decision-making space?**

Why this consideration is important

Participants’ entry point into a decision-making process does not exclusively determine the quality of their participation. A variety of *levels* of participation exist, with some more active and influential than others. Sherry Arnstein (1969) and others distinguish between the following levels of participation: collaboration, consultation, and information-giving.

*Collaboration* involves shared decision-making between the research team and community members. Both parties deliberate together and their knowledge is integrated during priority-setting. The final research topic and questions are an explicit product of deliberation that is endorsed by all participants. They are a ratified collective conclusion and reflect the voices of the research team and community members.

*Consultation* is characterised by community members being invited to give their input through interviews or focus groups but having no assurance that it will be used by researchers. This gives the research team the power to interpret the content of interviews, focus groups, or deliberations amongst community members. They construct possible research topics and questions using that content and decide which to study. Here, the final research topic and questions are an inferred product constructed and selected by the research team.

*Informing* means community members are told of the outputs of priority-setting for research projects but do not have a say in the process or decisions in any way.

Community members’ level of participation can vary or remain consistent amongst the four stages of the priority-setting process.

Some options for constructing engagement in priority-setting are listed below that reflect these levels of participation. Multiple or a single option can be used in a single priority-setting process. The first two options are collaborative; the research team *and* community members identify possible research topics and/or questions and decide which to study. In the latter three consultative options, the research team does so alone.

1. Whole-group deliberative discussion.
2. Hold separate deliberative small group discussions for different groups and then bring everyone together for a whole-group deliberative discussion.
3. Consult all community members in single deliberative group discussion.
4. Consult community members in separate deliberative small group discussions.
5. Consult community members individually through interviews.

Power is more evenly shared where community members participate in health research priority-setting as collaborators or decision-makers—Options 1 and 2 above. Deliberative processes are more likely to bring different ways of knowing and perspectives together to create outputs that reflect a diversity of people’s knowledge.

BUT under some circumstances, Options 1 and 2 may not be appropriate. It may not be *fair* to bring certain community members into the same decision-making space. Three examples of such circumstances are provided:

- Oppressor situation- Certain community members are responsible for oppressing other community members. The oppressors and/or the community members they oppress will require a separate consultation.
- Power disparities situation- Power disparities are too large to mitigate sufficiently between certain community members. Those community members who are unlikely to raise their voice in the presence of others may require separate consultations.
- Missing foundations situation- Respect and trust is lacking between community members. Certain community members may treat others in a very disrespectful manner when in the same space. Without trust, certain community members may not feel comfortable sharing their ideas and perspectives in front of others. Without skills for negotiation or knowledge of research processes, it will be very difficult for some community members to hold their own in a decision-making space with others who have greater negotiation and research literacy. To avoid harms of disrespect and to ensure the voices of all participants are heard may require performing separate consultations.

It is thus important to assess whether it is fair to bring particular representatives of community roles into the same decision-making space. In some instances, Options 3, 4, or 5 above may be the more appropriate way to access their voices. Power can still be shared with the community in Options 3-5, particularly where community members are consulted by field investigators from the community. And where data from consultations is analysed by research team members that include field investigators, community partners, and locally-based researchers.

Who within the community are cast as collaborators (decision-makers) versus consultants also helps determine the extent to which engagement reinforces or challenges perceived power inequalities amongst community members. For example, researchers may reinforce existing power dynamics within communities if they collaborate with community leaders and consult with non-aligned community members.

**6. REPRESENTATION**

**6a. Which organisations or individuals will represent the roles listed in Q3a?**

**6b. Do these representatives encompass the disadvantaged, less influential, lower status, or marginalised within each role, as identified in Question 3c?**

Why this consideration is important

Once roles to include are identified, determining who should represent them is essential. Two types of representatives are: 1) organisations and 2) individuals.

Selected representatives should genuinely reflect the needs and interests of the role they are representing in a way that’s accountable. They should also encompass the disadvantaged, less influential, lower status, and/or marginalised within each role to ensure that participants in priority-setting reflect the community’s diversity.

Desirable features of organisational representatives include their having a systemic practice of consulting members on their needs and priorities, having a systemic practice of reporting back to members, and having members who reflect the diversity of the role or group it represents. For patient organisations, this could mean having members that span different genders, levels of education, ethnicities or castes, levels of income, residences (urban versus rural), and employment statuses (formal versus informal).

Desirable features of individual representatives include living a similar reality to those they are representing; being able to think from the perspective of others in their community or with their role, particularly those who are of lower status or considered disadvantaged; and being able and willing to relay information back to those they represent.

**7. mass**

**7a. Will the number of representatives of lower status community roles be equal to or exceed the number of representatives of higher status community roles at each stage of the priority-setting process? If not, what are your reasons?**

**7b. Will a sufficient number of representatives of stakeholders identified in Question 3c be engaged in each stage of the priority-setting process? If not, what are your reasons?**

Why this consideration is important

To ensure that the powerful do not dominate priority-setting by force of numbers, it is necessary to balance power through slight inequalities in numbers. Although lower status roles will vary by setting, this might, for example, entail having equal or more patient and non-aligned community member representatives to policymaker and community leader representatives.

Achieving sufficient numbers of stakeholders who are considered disadvantaged, less influential, lower status, and/or marginalised is also essential. What this looks like may vary based on the type of stakeholder. For instance, in communities where women are considered less influential or lower status than men, it could mean having equal or greater numbers of women relative to men present at each stage of the priority-setting process. In a community where leaders from remote sub-districts are less influential, it would be important to ensure equal numbers of leaders from each sub-district, possibly with more leaders from remote sub-districts in attendance.

**8. community assets**

**What assets within the community can be used to help recruit its members, especially those considered disadvantaged, less influential, lower status, and/or marginalised, and to bring out their voices during priority-setting?**

Why this consideration is important

Communities have assets that can facilitate power-sharing in health research priority-setting. These assets can help ensure community members are represented and their voices are heard in priority-setting. They encompass physical and human resources, information and skills, networks, and participatory structures:

- Physical: Spaces where priority-setting can be held
- Information and skills: Locally-based researchers who can help lead research projects; community champions or key informants who can provide information about the community and help with recruitment for priority-setting; peer leaders, community workers or others skilled in facilitation; community members with prior experience of research; and community members with experiential knowledge of particular topics
- Networks: Community partners’ networks within the community can help facilitate recruitment, especially of those considered disadvantaged and marginalised, to priority-setting
- Participatory structures: Structures designed to give voice to community members like community dialogues can be utilised in priority-setting

It is important such assets be used in priority-setting and that communities are appropriately compensated for their use. Otherwise, you may be draining a community’s resources, which could be detrimental to its members (see Key Consideration: Resources and Compensation)

**9. space**

**Where will you hold the priority-setting process for your research project?**

Why this consideration is important

The physical spaces selected for health research priority-setting can promote or obstruct the presence and voice of community members, particularly those considered marginalised and disadvantaged. Where spaces are physically difficult for community members to access, it can result in their not being present for priority-setting. Where spaces are imbued with certain norms, behaviours, and languages, people favoured by those norms or more practiced in those behaviours and languages will dominate priority-setting. If priority-setting processes are held at research institutions or the Ministry of Health, for example, community members may be less likely to raise their voices.

Holding priority-setting in spaces within the community can thus promote giving voice to its members. Nevertheless, local spaces may also be characterised by exclusionary norms, for example, where minorities are simply informed and generally do not have a say. Priority-setting using such spaces will likely mean those considered disadvantaged and marginalised within the community are present but rarely speak.

**10. framing**

**Will you make it clear to participants that any or most health research topics can be raised during priority-setting? [Worksheet 3A]**

**OR**

**Will it been made clear to participants that *not all* health research topics can be raised during health research priority-setting and why that is? [Worksheet 3B]**

Why this consideration is important

To frame an issue means to select and highlight certain aspects of it and is a key site of power in decision-making. Whoever controls the frame of an issue shapes the outcome of the decision-making process. In health research priority-setting, framing can occur when the topics that can be raised are narrowed from the start, distorting or restricting what and/or whose health needs can be proposed as priorities. For example, if individual-level causes (biological traits, individual behaviour) are emphasised as the main drivers of poor health during priority-setting, this may exclude discussion of research topics related to population-level causes (environmental and social determinants).

Power is more likely to be shared when there is an “open” scope to set health research topics with community members: no or very few topics related to health are off the table. Where existing funding (or other) constraints make this impossible, it is essential to be transparent about it at the start of priority-setting. Transparency can help prevent community members from developing unrealistic expectations about what the priority-setting process can deliver. Where there is NOT an “open” scope to set research topics and questions with community members, it must be made very clear to participants what topics are off the table and why that is *before* starting the priority-setting process.

**11. ground rules**

**11a. Will you involve community members in developing and approving the ground rules for priority-setting? If not, what are your reasons?**

**11b.** **What ground rules will you include to ensure stakeholders identified in Question 3c aren’t silenced during priority-setting?**

**11c. How will ground rules be clearly communicated to participants in priority-setting?**

Why this consideration is important

What ground rules are set for the priority-setting process have a significant impact on who is present and whether their voices can be raised and heard. Ground rules specify who can and can’t be participants, who can speak, what languages are used, how different participants’ views are used, and how a decision or closure is reached.

Power is more likely to be shared where ground rules are developed with community members and they promote the inclusion of marginalised groups in priority-setting. For example, you might include ground rules that privilege ways of speaking like storytelling and rhetoric, affirm that everyone has an equal right to speak, and clearly define how community members’ views and ideas will be used to set research topics and questions.

Transparency is important to building community members’ trust in the research team and the legitimacy of the health research priority-setting process. Ensuring its ground rules are disclosed and explained to community members can promote their understanding of how the process will work and thus their capacity to raise their voices.

**12. facilitation**

**12a. Will you have a locally-based person facilitate deliberations during priority-setting? If not, what are your reasons?**

**12b. How will the facilitation method/approach help equalise power dynamics between community members?**

Why this consideration is important

Good facilitation is critical because priority-setting spaces are not neutral. Asymmetrical power dynamics exist within them, both between researchers and community members and amongst community members. Such power dynamics can result in certain individuals being excluded from raising their voice and being heard. For example, higher status may be afforded to expert knowledge over local knowledge, giving greater weight to the voices of researchers, policymakers, and practitioners over patients, carers, and other community members.

A certain approach to facilitation is necessary to make sure that the least powerful voices get as much opportunity to be heard as the most powerful voices during deliberations. Very careful set up is necessary in most contexts for representatives of lower status community roles to raise their voices in priority-setting.

**13. documentation**

**13a. Will you have a locally-based person document the priority-setting process? If not, what are your reasons?**

**13b. How will community members be given an opportunity to review the documentation of the priority-setting process?**

Why this consideration is important

Documentation of what is said in health research priority-setting can ensure that the voices of community members, including those considered disadvantaged and marginalised, are captured. It can promote their being heard because their comments and views are written down or recorded.

Yet, like facilitation, documentation of priority-setting must be done in a particular way to share power with community members. It should be performed by community members rather than research staff. Its outputs should be made available to participants in local languages and a mechanism for participants to review them should exist.

**14. synthesis**

**Will you give the voices of consulted community members, especially those considered disadvantaged, less influential, lower status, and/or marginalised, equal or greater weight than other voices when setting research priorities? If not, what are your reasons?**

Why this consideration is important

Where research priorities are set after consultations, whose knowledge is used to set them and who controls the integration of that knowledge determine what outputs emerge from priority-setting. Here, members of the research team will typically control the translation of knowledge gained from consultations with community members into research priorities. When doing so, it is essential that they use the views expressed by community members, especially those considered disadvantaged and marginalised, to construct the given project’s research topic and questions. Otherwise, community members’ voices won’t be heard.

**15. Resources and compensation**

**15a. How will communities be compensated for the use of their assets during priority-setting?**

**15b. Will community partners have control over any project resources?**

**15c. Will full information about the research project’s budget be disclosed to community partners?**

Why this consideration is important

It is essential to compensate communities for use of their assets during priority-setting. Priority-setting processes can be time-consuming and involve significant amounts of work and it is unfair and disrespectful to expect key informants, field investigators, community researchers, facilitators, and community members who participate in priority-setting to contribute without compensation. It is also less likely that these parties will be able to participate in priority-setting without compensation because the lost work hours and income will be too significant for it to be feasible.

For community members, compensation should cover but not exceed their time and transport. In some instances, where community members participate during work hours for which they receive a salary, they may not need or want compensation and it is important to ask them whether they do. It may not be necessary to provide compensation in such cases. For field investigators and community researchers, best practice means paying them as per a normal job, e.g. through the research institution’s normal human resources procedure for casual academic contracts.

Additionally, sharing power in priority-setting means sharing control over project resources and being transparent about them with community partners. Where community partners are leading activities or stages of priority-setting, they should be given project resources to spend to carry out them out. To achieve transparency, community partners should also be given a copy of the research project’s budget and given an explanation of what the different line items in it mean. Involving community partners in setting the research project budget to begin with is another important way to share power with them.

**16. Unintended harms**

**What harms do you think might result from the priority-setting process?**

Why this consideration is important

Health research priority-setting processes that engage community members have the potential to generate harms for them and community partners. It is, therefore, important to try and anticipate what harms could result from the priority-setting process. If significant harms are identified, revisions to the priority-setting process design should be made. Examples of possible harms that could eventuate from a priority-setting process are:

- Disruption of community power dynamics leads to negative repercussions for members of already stigmatised and marginalised groups.
- The safety of certain groups with illegal status is compromised.
- Some community members experience disrespect from other community members during priority-setting.
- Community members are labelled as marginalised or having lower status roles.
- Community members are taken away from work or other responsibilities for too much time.
- The community partner’s relationships with community leaders and/or members are negatively affected.
- Community members develop unrealistic expectations about what the research can deliver.

**17. Respect**

**How will you demonstrate respect to community members and their culture during priority-setting?**

Why this consideration is important

Demonstrating respect is a key way to promote the presence and voice of community members, including those considered disadvantaged and marginalised, in health research priority-setting. Two forms of respect are important here: interpersonal and cultural. Interpersonal respect means treating people as though they and their contributions to the priority-setting process are equally valued. This helps make community members feel comfortable expressing their views. Interpersonal respect also means making sure people don’t feel used, e.g. that you’re not involving them just to rubber stamp what you’re already planning to do.

Demonstrating cultural respect means showing you value local cultural norms and following them as much as possible (or asking if something is acceptable if you are not sure). For example, where local ways of speaking (e.g. types of greetings, storytelling) are immediately shut down during priority-setting, it can be very disrespectful and make community members hesitant to raise their voices. Demonstrating cultural respect also means showing consideration for local power dynamics as much as possible.

**18. accountability**

**18a. How will you feed back the final research topic and questions to community members, including those considered to be marginalised, after priority-setting?**

**18b. How** **will you act upon the final research topic and questions?**

**18c.** **How are you and community members going to evaluate their engagement in the priority-setting process?**

**18d. How will community engagement continue during the research project and after it finishes?**

Why this consideration is important

Accountability is important for ensuring community members’ voices are heard and building the legitimacy of the priority-setting process. It means that participants and their community have the right to hold the research team to a set of standards, to judge whether the research team has fulfilled its responsibilities, and to take measures if they determine that these responsibilities have not been met.

Three responsibilities of the research team are: 1) feeding back, 2) acting on research priorities, and 3) evaluation. Feeding back means sharing resultant research priorities with participants in the process and giving them an opportunity to comment on the priorities. Achieving action on the research priorities ensures that community members’ voices are heard. It means that research projects focusing on the identified priorities are funded and performed. Finally, accountability as evaluation means developing benchmarks for power-sharing in the engagement process and assessing whether they were met after priority-setting is completed. Benchmarks could include achieving a diversity of participants and getting people’s voices heard. Where evaluations demonstrate that community engagement in priority-setting fell far short of power-sharing, it then raises questions about whether it is ethical to take resultant research priorities forward.
