# Supplementary material for: Developing a toolkit for engagement practice: sharing power with communities in priority-setting for global health research projects
Source: BMC Med Ethics. 2020 Mar 14;21:21. doi: 10.1186/s12910-020-0462-y (PMC7071780; doi:10.1186/s12910-020-0462-y)
Supplement: Supplementary file 2 — Additional file 2. Sharing Power with Communities in Priority-Setting for Health Research Projects: A Toolkit. Worksheet 1. [file 12910_2020_462_MOESM2_ESM.docx]

Bridget Pratt

Identifier first line

- - Second line

**Selecting Partners Worksheet:**

**Question for Reflection and Discussion**

This worksheet should be completed by the research team collectively. Please read the Companion Document: Key Considerations in Worksheet 1. To complete Worksheet 1, reflect on and discuss Question 1 as a team. Record your team’s answer and read the Next Steps to take. Finally, identify Strategies and/or Actions to Take and record them below.

**Question 1:** **Does your research team include a research partner(s) and a community partner(s) who can access a community that is considered disadvantaged and/or marginalised?**

| Team Answer |
| --- |

| Next steps   - If your answer is yes, move to Worksheet 2. - If your answer is no, brainstorm possible candidate researchers or research institutions to partner with that have the requisite values, capacities and expertise to conduct research with communities considered to be disadvantaged or marginalised. Identify Strategies and/or Actions to Take to reach out to top candidates. - If your answer is no, brainstorm possible candidate community-based organisations to partner with that have the requisite values, capacities and standing to access the voices of communities considered to be disadvantaged or marginalised. Identify Strategies and/or Actions to Take to reach out to top candidates. Draw on key informants’ knowledge and suggestions when compiling a list of candidate organisations and when determining how best to reach out to them. If no appropriate community-based organisations can be identified in the community, move on to Worksheet 2. |
| --- |

| Strategies and/or Actions to Take |
| --- |
| Strategies and/or Actions to Take cont’d |
