# Supplementary material for: Developing a toolkit for engagement practice: sharing power with communities in priority-setting for global health research projects
Source: BMC Med Ethics. 2020 Mar 14;21:21. doi: 10.1186/s12910-020-0462-y (PMC7071780; doi:10.1186/s12910-020-0462-y)
Supplement: Supplementary file 3 — Additional file 3. Sharing Power with Communities in Priority-Setting for Health Research Projects: A Toolkit. Worksheet 2. [file 12910_2020_462_MOESM3_ESM.docx]

Identifier first line

- - Second line

Bridget Pratt

**Deciding to Engage Worksheet:**

**Questions for Reflection and Discussion**

This worksheet should be completed by the research team collectively. Please first read the Companion Document: Key Considerations in Worksheet 2 and read the Deciding to Engage Worksheet Flowchart (p. 2) in order to understand how the questions in this worksheet relate to one another. Then complete the worksheet as a team. To start, reflect on and discuss Question 1 collectively. Record your team’s answer and read the Next Steps to take. Where the Next Steps ask you to identify Strategies and/or Actions to Take, do so as a team and record them before moving on to the next question in the worksheet.


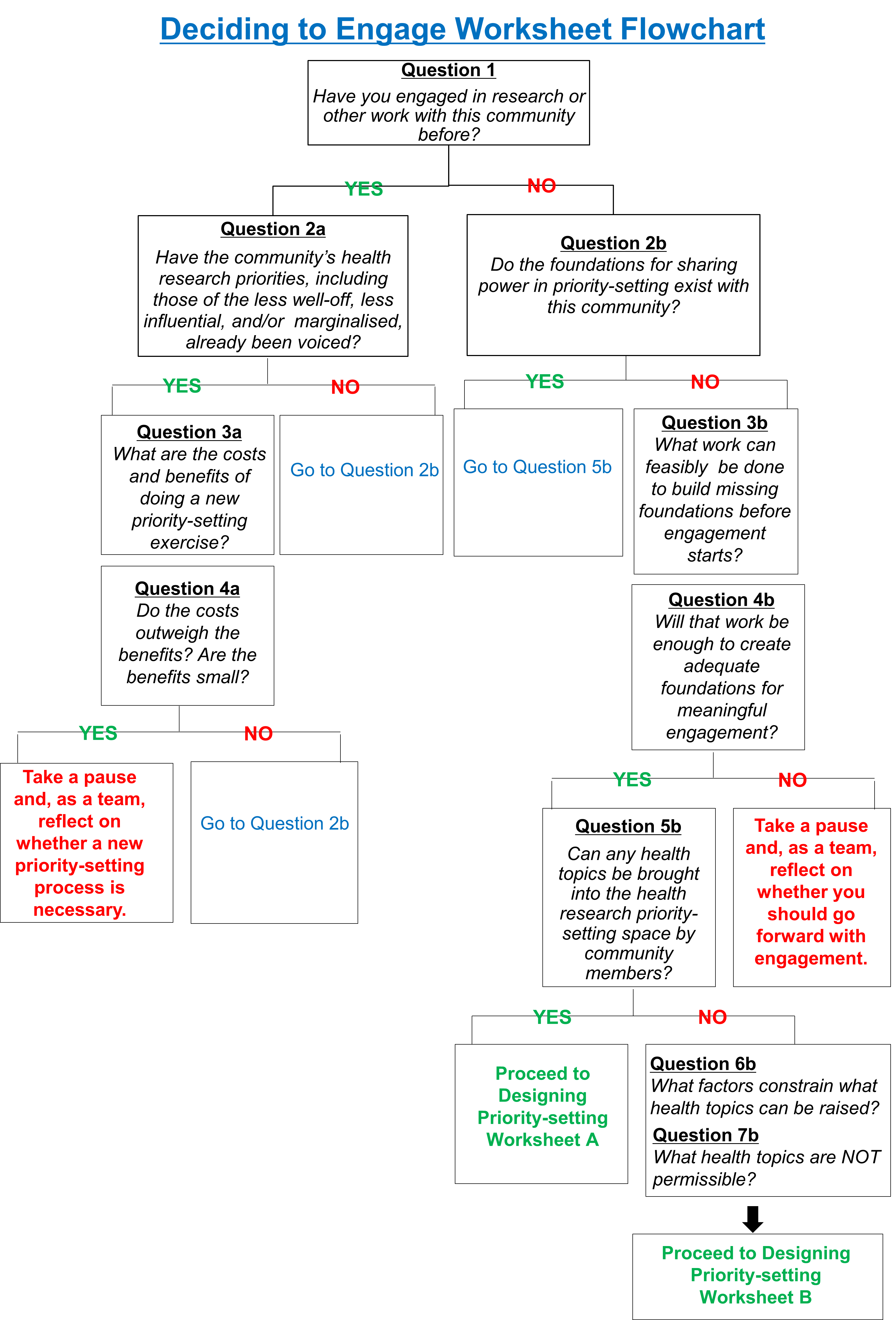


**Question 1: Have you engaged in research or other work with this community before?**

| Team Answer |
| --- |

| Next steps  If your answer is yes, complete Question 2a as a research team.  If your answer is no, complete Question 2b (page 4) as a research team. |
| --- |

**Question 2a: Have the community’s health research priorities, including those of the less well-off, less influential, and/or marginalised, already been voiced?**

| Team Answer |
| --- |

| Next steps  If your answer is yes, complete Question 3a as a research team.  If your answer is no, complete Question 2b (page 4) as a research team. |
| --- |

**Question 3a: What are the costs and benefits of doing a new priority-setting exercise?**

| Team Answer  Costs Benefits |
| --- |

| Next steps  Complete Question 4a as a research team. |
| --- |

**Question 4a: Do the costs outweigh the benefits? Are the benefits small?**

| Team Answer |
| --- |

| Next steps  If your answer is yes, take a moment to step back and reflect on whether a new priority-setting exercise is necessary. If the research team decides not to undertake a new priority-setting exercise, consider getting community approval on the (previously identified) topic before starting the new research project. If multiple research priorities were previously identified, the community may prefer researchers study certain topics before others. Brainstorm how you will obtain community approval and/or ranking of previously identified priorities under Strategies and/or Actions to Take.  If your answer is no, complete Question 2b (page 4) as a research team. |
| --- |

| Strategies and/or Actions to Take |
| --- |

**Question 2b: Do the foundations for sharing power in priority-setting exist with this community? To answer, assess for the following foundations:**

| Team Answer  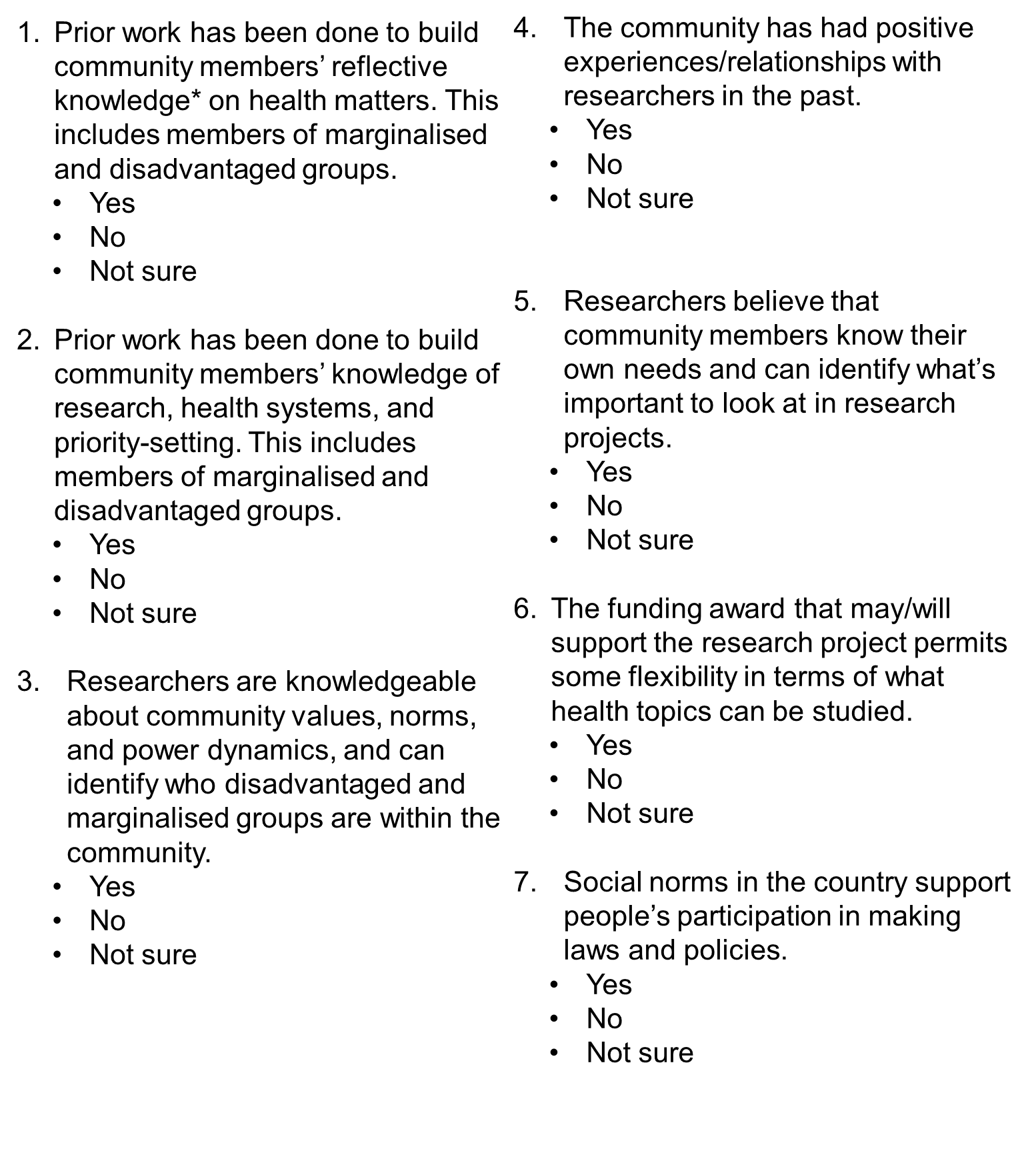 |
| --- |

* Reflective knowledge is defined in the Companion Document.

| Next steps  If your answer is yes for 6-7 foundations, complete Question 5b as a research team.  If your answer is no or not sure for 2 or more foundations, complete Question 3b as a research team. |
| --- |

**Question 3b: What work can feasibly be done to build missing foundations before engagement starts?**

**For each missing foundation, brainstorm strategies for developing it, e.g. trust-building activities, that are achievable for the research team. When doing so, consider what resources are available to implement each strategy.**

| Team Answer |
| --- |

| Next steps  Complete Question 4b as a research team. |
| --- |

**Question 4b: Will that work be enough to create adequate foundations for meaningful engagement?**

| Team Answer |
| --- |

| Next steps  If your answer is yes, complete Question 5b as a research team.  If your answer is no, take a moment, as a team, to reflect on whether you should go forward with engagement. |
| --- |

**Question 5b: Can any health topics be brought into the health research priority-setting space by community members?**

| Team Answer |
| --- |

| Next steps  If your answer is yes, proceed to Worksheet 3A.  If your answer is no, complete Questions 6a and 7a as a research team. |
| --- |

**Question 6a: What factors constrain what health topics can be raised?**

| Team Answer  Funding constraints:  Researcher expertise constraints:  Other: |
| --- |

**Question 7a: What health topics are NOT permissible?**

| Team Answer |
| --- |

| Next steps  Proceed to Worksheet 3B. |
| --- |
