# Supplementary material for: Developing a toolkit for engagement practice: sharing power with communities in priority-setting for global health research projects
Source: BMC Med Ethics. 2020 Mar 14;21:21. doi: 10.1186/s12910-020-0462-y (PMC7071780; doi:10.1186/s12910-020-0462-y)
Supplement: Supplementary file 5 — Additional file 5. Sharing Power with Communities in Priority-Setting for Health Research Projects: A Toolkit. Worksheet 3B. [file 12910_2020_462_MOESM5_ESM.docx]

Bridget Pratt

Identifier first line

- - Second line

**Designing Priority-setting Worksheet:**

**Questions for Reflection and Discussion**

This worksheet should be completed by the research team collectively. Please first read the Companion Document: Key Considerations in Worksheet 3 and the summary of the questions in this worksheet below.

After reading both, complete Worksheet 3 as a team. Before answering each question, read the Points to Consider (if applicable). Then reflect on and discuss the question collectively. Record your team's answer and read the Next Steps to take. Where the Next Steps ask you to brainstorm Strategies and/or Actions to Take, do so as a team and record them before moving on to the next question in the worksheet.

If you find that you are unable to answer many of the questions in this worksheet, as a team, consider returning to the Deciding to Engage Worksheet and further reflecting on whether the foundations for meaningful engagement are present and/or how to further strengthen them.

| **Summary of Worksheet 3A Questions for Reflection and Discussion**  Leadership  1. Who will initiate and lead engagement with community members during health research priority-setting?  Empowerment  2. Will community partners' and members' capacities be strengthened during priority-setting?  Diversity within the community  3a. Which community roles will you engage during priority-setting and for what reasons?  3b. List which of the roles identified in Q3a correspond to greater or lesser  influence and status within the community.  3c. Who are considered disadvantaged, less influential, lower status, or marginalised within these roles?  3d. Which of these groups or stakeholders will you engage and for what reasons?  Stage of Participation  4a. Will you involve community partners and members from the start of the priority­ setting process? If not, what are your reasons?  4b. Will less influential and lower status community roles be involved later and in fewer stages of the priority-setting process than higher status and more influential roles?    Level of Participation  5a. Will you involve community members as collaborators (decision-makers) in priority-setting? If yes, in what stages of the priority-setting process? If not, what are your reasons?  5b. Is it fair to bring these community members into the same decision-making space?  Representation  6a. Which organisations or individuals will represent the roles listed in Q3a?  6b. Do these representatives encompass the disadvantaged, less influential, lower status, and/or marginalised within each role, as identified in Question 3c?  Mass  7a. Will the number of representatives of lower status community roles be equal to or exceed the number of representatives of higher status community roles at each stage of the priority-setting process? If not, what are your reasons?  7b. Will a sufficient number of representatives of stakeholders identified in Question 3c be engaged in each stage of the priority-setting process? If not, what are your reasons?  Community Assets  8. What assets within the community can be used to help recruit its members, especially those considered disadvantaged , less influential, lower status, and/or marginalised , and to bring out their voices during priority-setting?  Space  9. Where will you hold the priority-setting process for your research project?  Framing  10. Will it been made clear to participants that not all health research topics can be raised during health research priority-setting and why that is?  Ground Rules  11a. Will you involve community members in developing and approving the ground rules for priority-setting? If not, what are your reasons?  11b. What ground rules will you include to ensure stakeholders identified in Question 3c aren't silenced during priority-setting?  11c. How will ground rules be clearly communicated to participants in priority­ setting?  Facilitation  12a. Will you have a locally-based person facilitate deliberations during priority­ setting? If not, what are your reasons?  12b. How will the facilitation method/approach health equalize power dynamics between community members?  Documentation  13a. Will you have a locally-based person document the priority-setting process? If not, what are your reasons?  13b. How will community members be given an opportunity to review the  documentation of the priority-setting process?  Synthesis  14. Will you give the voices of consulted community members, especially those considered disadvantaged, less influential, lower status, and/or marginalised, equal or greater weight than other voices when setting research priorities? If not, what are your reasons?  Resources and Compensation  15a. How will communities be compensated for the use of their assets during priority-setting?  15b. Will community partners have control over any project resources?  15c. Will full information about the research project's budget be disclosed to community partners?  Unintended harms  16. What harms do you think might result from the priority-setting process?  Respect  17. How will you demonstrate respect to community members and their culture during priority-setting?  Accountability  18a. How will you feed back the final research topic and questions to community members, including those considered to be marginalised, after priority-setting?  18b. How will you act upon the final research topic and questions?  18c. How are you and community members going to evaluate their engagement in the priority-setting process?  18d. How will community engagement continue during the research project and after it finishes? |
| --- |

**1. Leadership**

**Who will initiate and lead engagement with community members during health research priority-setting?**

Points to consider:

1. Does your choice help reduce perceived inequalities in power between researchers and community members?

| Team Answer |
| --- |

| Next steps  Where those initiating engagement do not include locally-based researchers, community partners, or key informants:   - Look for locally-based or Indigenous researchers who are known to and trusted by the community to add as principal investigators or co-investigators. - Develop a plan for approaching candidates about joining the research team. - Discuss with community partners whether they have staff with the interest and capacity to be a principal or co-investigator. |
| --- |

| Strategies and/or Actions to Take |
| --- |

**2. EMPOWERMENT**

**2. Will community partners’ and members’ capacities be strengthened during priority-setting?**

| Team Answer |
| --- |

| Next steps  If your answer is yes, brainstorm *how* the priority-setting process can build the knowledge, confidence, networks, and/or skills of community partners and members. Draw on key informants’ insights and recommendations when developing these strategies.  If your answer is no and the purpose of engagement is solely instrumental, it should be made transparent to those engaged and justified to them. Discuss how this will be done as a research team. |
| --- |

| Strategies and/or Actions to Take |
| --- |

**3. diversity within the community**

**3a. Which community roles will you engage during priority-setting and for what reasons?**

**3b. List which of the roles identified in Q3a correspond to greater or lesser**

**influence and status within the community.**

**3c. Who are considered disadvantaged, less influential, lower status, or marginalised within these roles?**

**3d. Which of these groups or stakeholders will you engage and for what reasons?**

Points to consider:

1. Could you undertake stakeholder mapping with key informants in order to identify what roles are present in the community?
2. Which of those roles are relevant to include as participants? For example, advancing values of equity and social justice speak to two main reasons for selecting particular community roles to involve in priority-setting: because they either have pertinent knowledge of the health needs of marginalised groups,
3. or because they have the power to change policies and practices that affect those groups' health.
4. Could you develop a definition of what constitutes 'disadvantage' or 'marginalised' in the community with key informants and use it to identify who are considered disadvantaged and/or marginalised within the relevant roles?

| Team Answer  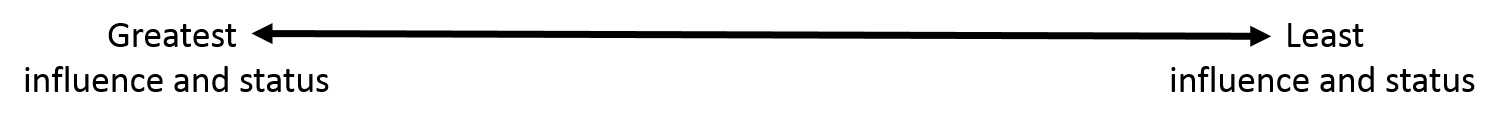 |
| --- |

| Team Answer  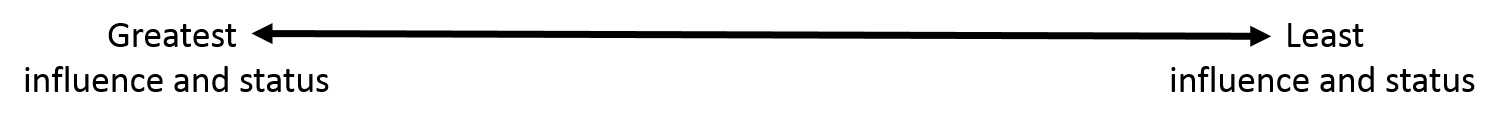 |
| --- |

**4. stage of participation**

**4a. Will you involve community partners and members from the start of the priority-setting process? If not, what are your reasons?**

**4b. Will less influential and lower status community roles be involved later and in fewer stages of the priority-setting process than higher status and more influential roles?**

| Team Answer |
| --- |

| Next steps  Fill in Worksheet 3 Supplemental Table, Column 2: ‘What community roles will be engaged?’  If your answer is yes, then proceed to Question 5.  If your answer to Question 4a and/or 4b is no, discuss how it might be possible to include community partners, community members, and/or marginalised groups earlier in the priority-setting process. |
| --- |

| Strategies and/or Actions to Take |
| --- |

**5. level of participation**

**5a. Will you involve community members as collaborators (decision-makers) in priority-setting? If yes, in what stages of the priority-setting process? If not, what are your reasons?**

**5b. Is it fair to bring these community members into the same decision-making space?**

| Team Answer |
| --- |

| Next steps   - Fill in Worksheet 3 Supplemental Table, Column 2: ‘What level of participation will each role have?’ - If your answer to Q5a is yes, consider whether and how research priorities can be set through a deliberative process with community members that yields a collective decision. Deliberative community engagement processes have been used to inform institutional ethics policies on biobanking and benefit sharing. Methods applied in these studies may be a rich resource to draw upon to inform health research priority-setting practice.^^[[1]](#footnote-1)^^ - If your answer to Q5a is no and research priorities will be decided by the research team after consultations, consider how a ratification process involving community members, including those considered marginalised, can be implemented. - In some contexts, having the research team set priorities after conducting community consultations may necessary because community members cannot be brought together in the same deliberative fora for ethical reasons or due to cultural norms. For example, in South Africa, local administrators can be powerful oppressors of the LGBTQ community and their access to health services. It would arguably not be desirable for them to participate in the same deliberative forum as members of LGBTQ community. Where ethical reasons or cultural norms make deliberations between certain community members (e.g. elders and youths) inappropriate, this may require undertaking separate consultations with them. |
| --- |

| Strategies and/or Actions to Take |
| --- |

**6. REPRESENTATION**

**6a. Which organisations or individuals will represent the roles listed in Q3a?**

**6b. Do these representatives encompass the disadvantaged, less influential, lower status, and/or marginalised within each role, as identified in Question 3c?**

Points to consider:

1. Does it make sense to ask community leaders or key informants to select individuals or organisations to represent the identified roles? If yes, consider giving them some criteria that you're hoping representatives will meet in order to avoid selection biases.
2. Do you have evidence that these organisations' memberships reflect the roles' diversity and are regularly consulted about their needs and priorities?
3. Where individuals will represent a role, do they collectively reflect its diversity and share lived experience with those they are representing?
4. Do any of the selected representatives have substantial financial conflicts of interest that you think will bias their identification of research priorities?

| Team Answer |
| --- |

| Next steps   - Fill in Worksheet 3 Supplemental Table, Columns 4 and 5 using your answers to Questions 6a and 6b. - As a research team, develop strategies to recruit identified representatives . One option could be to ask community leaders or key informants to assist with recruiting them, particularly those who may be hard to reach. Another option is to discuss how your community partner can facilitate the recruitment of identified representatives. |
| --- |

| Strategies and/or Actions to Take |
| --- |

**7. mass**

**7a.Will the number of representatives of lower status community roles be equal to or exceed the number of representatives of higher status community roles at each stage of the priority-setting process? If not, what are your reasons?**

**7b. Will a sufficient number of representatives of stakeholders identified in Question 3c be engaged in each stage of the priority-setting process? If not, what are your reasons?**

| Team Answer |
| --- |

| Next steps   - Fill in Worksheet 3 Supplemental Table, Columns 6 and 7 - If your answer to Q6b is yes, proceed to Q7. - If your answer to Q6b is no, brainstorm with key informants whether there are additional organisations or individuals who could represent lower status roles. Develop strategies to recruit them to participate in priority-setting. |
| --- |

| Strategies and/or Actions to Take |
| --- |

**8. community assets**

**What assets within the community can be used to help recruit its members, especially those considered disadvantaged, less influential, lower status, and/or marginalised, and to bring out their voices during priority-setting?**

| Team Answer |
| --- |

| Next steps  As a research team, brainstorm strategies for how to access to the identified community assets. Draw on key informants’ insights and recommendations when doing so. |
| --- |

| Strategies and/or Actions to Take |
| --- |

**9. space**

**Where will you hold the priority-setting process for your research project?**

Points to consider:

- Is the space you have selected physically accessible and safe for those considered disadvantaged and marginalised within the community? Draw on key informants’ knowledge to make this assessment.
- What norms are associated with the space? Do these norms silence those considered marginalised? Draw on key informants’ knowledge to make this assessment.

| Team Answer |
| --- |

| Next steps  If the chosen space is physically accessible, safe, and not imbued with norms that will silence marginalised groups, develop a plan for gaining permission to use it for your priority-setting process.  If the space does not meet those criteria, brainstorm other possible locations that do and develop a plan for gaining permission to use one of them. |
| --- |

| Strategies and/or Actions to Take |
| --- |

**10. framing**

**Will it been made clear to participants that *not all* health research topics can be raised during health research priority-setting and why that is?**

| Team Answer |
| --- |

| Next steps  If your answer is yes, brainstorm how to explain to participants (at the start of priority-setting) that not all health topics can be proposed during priority-setting and why that is the case.  If your answer is no, discuss your reasons for the lack of transparency and how you can avoid raising unrealistic expectations for what the research can deliver. |
| --- |

| Strategies and/or Actions to Take |
| --- |

**11. ground rules**

**11a. Will you involve community members in developing and approving the ground rules for priority-setting? If not, what are your reasons?**

**11b. What ground rules will you include to ensure stakeholders identified in Question 3c aren't silenced during priority-setting?**

**11c. How will ground rules be clearly communicated to participants in priority­ setting?**

| Team Answer |
| --- |

| Next steps  As a research team, develop a plan to set ground rules for the priority-setting process. To help involve community members, including those considered disadvantaged and marginalised, in selecting ground rules, the following steps are suggested:   - Establish an initial list of ground rules and circulate it to participants, giving them time to comment. Then revise the list and get it approved by all participants *before* priority-setting starts. - When developing the initial list of ground rules, ask key informants what rules are essential to ensure members of marginalised groups have an equal opportunity to share their views during priority-setting. |
| --- |

| Strategies and/or Actions to Take |
| --- |

**12. facilitation**

**12a. Will you have a locally-based person facilitate focus groups or deliberations during priority-setting?** **If not, what are your reasons?**

**12b. How will the facilitation method/approach help equalise power dynamics between community members?**

Points to consider:

- Would a “stepped” approach be appropriate? In a “stepped” approach, small groups with some degree of homogeneity or similar characteristics deliberate first before everyone (or representatives of each small group) comes together as a large group. Such an approach helps reduce the impact of power disparities between groups by giving those who might otherwise be silenced a safe space to express themselves and to reflect on their ideas before having to present them to a wider audience.
- Can deliberations incorporate the use of local languages and ways of speaking like storytelling, pictures, dramas, and songs?

| Team Answer |
| --- |

| Next steps  As a research team, brainstorm who from the community or community partner has a strong understanding of community members’ relationships and hierarchies, speaks local languages, and is acceptable and trusted by participants and, therefore, would make a good facilitator. Develop a plan with key informants to approach the top candidates. |
| --- |

| Strategies and/or Actions to Take |
| --- |

**13. documentation**

**13a. Will you have a locally-based person document the priority-setting process? If not, what are your reasons?**

**13b. How will community members be given an opportunity to review the documentation of the priority-setting process?**

| Team Answer |
| --- |

| Next steps  As a research team, brainstorm who within the community or CBO partner could be asked to document the priority-setting process. Develop a plan with key informants to approach the top candidates.  As a research team, brainstorm how members of marginalised groups can be given a fair opportunity to review the documentation of the priority-setting process. |
| --- |

| Strategies and/or Actions to Take |
| --- |

**WHERE the final research priorities will be an explicit product of deliberation between the research team and community members, please skip Question 14.**

**14. synthesis**

**Will you give the voices of consulted community members, especially those considered disadvantaged, less influential, lower status, and/or marginalised, equal or greater weight than other voices when setting research priorities? If not, what are your reasons?**

| Team Answer |
| --- |

| Next steps  If your answer is yes, brainstorm what might comprise reasonable rationales for giving certain community members' voices greater weight than others in health research priority-setting. Many voices, including opposing ones, will be raised in interviews, focus groups, and deliberations and this will create a dilemma of whose voices to take forward and whose to drop. In practice, rationales that have been used include: amplifying unheard voices, amplifying voices that will create change, amplifying voices based on the values they further, and avoiding reinforcing negative stereotypes of certain groups.  If your answer is no, plan to be transparent and brainstorm how you will clarify this decision and the reasons behind it to participants. |
| --- |

| Strategies and/or Actions to Take |
| --- |

**15. resources and compensation**

**15a. How will communities be compensated for the use of their assets during priority-setting?**

**15b. Will community partners have control over any project resources?**

**15c. Will full information about the research project's budget be disclosed to community partners**?

| Team Answer |
| --- |

**16. Respect**

**How will you demonstrate respect to community members and their culture during priority-setting?**

Points to consider:

- How will you express and show community members that their views and contributions to priority-setting are valued?
- How will you design the priority-setting process to be considerate of community members’ time and hierarchy of needs?
- What cultural norms exist within the community and how can they be reflected in the priority-setting processes’ design?
- Can key informants help you better answer these questions?

| Team Answer |
| --- |

| Next steps  As a research team, brainstorm how you can show consideration for local power dynamics that may oppose your efforts to draw out the voices of marginalised groups in priority-setting. Discuss to what extent such power dynamics will or should be respected by the research team. |
| --- |

| Strategies and/or Actions to Take |
| --- |

**17. Unintended harms**

**What harms do you think might result from the priority-setting process?**

| Team Answer |
| --- |

| Next steps  As a research team, develop strategies to avoid or minimise the harms you have identified. Draw on key informants insights and recommendations when doing so. |
| --- |

| Strategies and/or Actions to Take |
| --- |

**18. accountability**

**18a. How will you feed back the final research topic and questions to community members, including those considered to be marginalised, after priority-setting?**

**18b. How will you act upon the final research topic and questions?**

**18c. How are you and community members going to evaluate their engagement in the priority-setting process?**

**18d. How will community engagement continue during the research project and after it finishes?**

| Team Answer |
| --- |

| Next steps  As a research team, discuss what you will do with any comments you receive from community members on the final research priorities.  As a research team, brainstorm how community members can be empowered to speak up and take action if they feel the research team hasn't met its responsibilities in terms of feeding back, acting on research priorities, and evaluation. |
| --- |

| Strategies and/or Actions to Take |
| --- |

1. See: O’Doherty, K.C., Hawkins, A.K., & Burgess, M.M. (2012). Involving Citizens in the Ethics of Biobank Research: Informing Institutional Policy through Structured Public Deliberation. *Social Science & Medicine*. 75, 1604-1611; Marsh, V. et al. (2013). Consulting Communities on Feedback of Genetic Findings in International Health Research: Sharing Sickle Cell Disease and Carrier Information in Coastal Kenya. *BMC Medical Ethics*. 14, 41; Njue, M., Kombe, F., Mwalukore, S., Molyneux, S., & Marsh, V. (2014) What Are Fair Study Benefits in International. Health Research? Consulting Community Members in Kenya. *PLoS* *ONE.* 9(12), e113112. doi:10.1371/journal.pone. 0113112 [↑](#footnote-ref-1)
