# Supplementary material for: Developing a toolkit for engagement practice: sharing power with communities in priority-setting for global health research projects
Source: BMC Med Ethics. 2020 Mar 14;21:21. doi: 10.1186/s12910-020-0462-y (PMC7071780; doi:10.1186/s12910-020-0462-y)
Supplement: Supplementary file 6 — Additional file 6. Sharing Power with Communities in Priority-Setting for Health Research Projects: A Toolkit. Worksheet 3 Supplemental Table. [file 12910_2020_462_MOESM6_ESM.docx]

|  | What community roles will be engaged? | What level of participation will each role have? | Which individuals or organisations will represent each role? | Do the representatives encompass identified categories of disadvantage, less influence, low status, and/or marginalisation within the role? | What is the total number of listed representatives for the community role? | What number of listed representatives correspond to each category of disadvantage, less influence, low status, and/or marginalisation within the role? |
| --- | --- | --- | --- | --- | --- | --- |
| Planning | Community leaders | Collaboration | Assume list of 10 individuals in this box | Women- Yes  Remote sub-districts- Yes | 10 | Women leaders- 5  Remote sub-district leaders- 5 |
|  |  |  |  |  |  |  |
|  |  |  |  |  |  |  |
|  |  |  |  |  |  |  |
|  |  |  |  |  |  |  |
|  |  |  |  |  |  |  |
|  | What community roles will be engaged? | What level of participation will each role have? | Which individuals or organisations will represent each role? | Do the representatives encompass identified categories of disadvantage, less influence, low status, and/or marginalisation within the role? | What is the total number of listed representatives for the community role? | What number of listed representatives correspond to each category of disadvantage, less influence, low status, and/or marginalisation within the role? |
| Topic Solicitation |  |  |  |  |  |  |
|  |  |  |  |  |  |  |
|  |  |  |  |  |  |  |
|  |  |  |  |  |  |  |
|  |  |  |  |  |  |  |
|  |  |  |  |  |  |  |
|  | What community roles will be engaged? | What level of participation will each role have? | Which individuals or organisations will represent each role? | Do the representatives encompass identified categories of disadvantage, less influence, low status, and/or marginalisation within the role? | What is the total number of listed representatives for the community role? | What number of listed representatives correspond to each category of disadvantage, less influence, low status, and/or marginalisation within the role? |
| Topic Prioritization |  |  |  |  |  |  |
|  |  |  |  |  |  |  |
|  |  |  |  |  |  |  |
|  |  |  |  |  |  |  |
|  |  |  |  |  |  |  |
|  |  |  |  |  |  |  |
|  | What community roles will be engaged? | What level of participation will each role have? | Which individuals or organisations will represent each role? | Do the representatives encompass identified categories of disadvantage, less influence, low status, and/or marginalisation within the role? | What is the total number of listed representatives for the community role? | What number of listed representatives correspond to each category of disadvantage, less influence, low status, and/or marginalisation within the role? |
| Setting Research Questions, Interventions |  |  |  |  |  |  |
|  |  |  |  |  |  |  |
|  |  |  |  |  |  |  |
|  |  |  |  |  |  |  |
|  |  |  |  |  |  |  |
|  |  |  |  |  |  |  |

Once you’ve filled out the worksheet, please consider the following:

1. Are less influential and lower status community roles included in early and most stages of the priority-setting process?

2. Are less influential and lower status community roles included as collaborators?

3. In each stage of priority-setting, do the number of representatives of less influential and lower status community roles equal or exceed the number of representatives of more influential and higher status community roles?
